# A novel feedback regulated loop of circRRM2-IGF2BP1-MYC promotes breast cancer metastasis

**DOI:** 10.1186/s12935-023-02895-w

**Published:** 2023-03-25

**Authors:** Ran Hao, Lei Zhang, Yangming Si, Peng Zhang, Yipeng Wang, Bangchao Li, Jie Hu, Yixin Qi

**Affiliations:** 1 Institutes of Health Research, Hebei Medical University, Shijiazhuang, Hebei China; 2 Department of Science and Technology, Hebei Medical University, Shijiazhuang, Hebei China; 3 Department of Breast Center, The Fourth Hospital of Hebei Medical University, Shijiazhuang, Hebei China; 4 School of Physical Science and Technology, Inner Mongolia University, Hohhot, Inner Mongolia China; 5 Department of Military Nursing, NCO School, Army Medical University, Shijiazhuang, Hebei China

**Keywords:** Circular RNA, Breast cancer, Metastasis, Feedback loop, MYC

## Abstract

**Background:**

Metastasis is the leading cause of mortality in patients with breast cancer (BC). Studies demonstrate that circular RNAs (circRNAs) were involved in BC progression, while the molecular mechanisms remain largely unclear.

**Methods:**

The microArray circRNA profiles were used to explore the differential expression circRNAs in BC and paracancerous normal tissues, and the quantitative reverse transcription-polymerase chain reaction was used to validate their expression level in clinical samples and cell lines. Nuclear/cytosolic fractionation and fluorescence in situ hybridization (FISH) assays were performed to examine circRRM2 (hsa_circ_0052582) subcellular location. The scratch wound healing and transwell assays were conducted to evaluate the impact of circRRM2 on BC cell migration and invasion. We predicted miRNAs that might bind with cricRRM2 and the downstream target genes using bioinformatics analysis and explored their expression levels and prognostic value in BC. FISH, RNA immunoprecipitation, Co-immunoprecipitation, Western blot, and rescue experiments were implemented to figure out circRRM2 function and underlying mechanisms in BC.

**Results:**

The present study revealed several aberrant circRNAs in BC tissues and observed that circRRM2 was upregulated in tumor tissues of 40 patients with BC. High circRRM2 was significantly associated with advanced N stage in patients with BC. Gain- and loss- of function experiments revealed that circRRM2 promoted the migration and invasion of cells and functioned as an oncogene in BC. Mechanism studies showed that circRRM2 competed with miR-31-5p/miR-27b-3p to upregulate the IGF2BP1 expression. Furthermore, IGF2BP1 upregulated the circRRM2 level via interacting with MYC, which functioned as the transcriptional factor of circRRM2. Thus, the positive feedback loop that was composed of circRRM2/IGF2BP1/MYC was identified.

**Conclusion:**

This study confirms that upregulated circRRM2 functions an oncogenic role in BC metastasis. The positive feedback loop of circRRM2/IGF2BP1/MYC enforces the circRRM2 expression, which might offer a potential target for BC treatment.

**Supplementary Information:**

The online version contains supplementary material available at 10.1186/s12935-023-02895-w.

## Introduction

Breast cancer (BC) is the most prevalent type of cancer in females, which causes predominant cancer-related mortality worldwide [1]. Despite significant progress in early diagnosis and comprehensive treatment, the prognosis of patients with BC continues to deteriorate [1]. Importantly, BC-related deaths are mainly caused by metastasis [2, 3]. However, little is known about the underlying mechanism by which cancer cells gain invasive capabilities during BC progression, which might represent a new strategy for BC metastasis prevention and treatment. In recent years, substantial evidence has revealed that non-coding RNAs (ncRNAs), including circular RNAs (circRNAs), are widely involved in BC progression [4, 5].

CircRNAs have covalently closed-loop structures, without a 5'-cap and a 3'-polyadenyl tail, and are highly conserved and stable [5]. circRNAs are generated by back-splicing or skipping precursor mRNAs in the eukaryotic genome [6]. Recent studies have revealed that circRNAs could impact various biological processes, including transcriptional regulation, protein translation, and immune regulation [6]. CircRNA plays a variety of roles, including competing endogenous RNA (ceRNA), interfacing with proteins, and being translated into proteins [7]. The association between malignant behavior and circRNAs has recently increased in BC. For instance, circSEPT9 was reported upregulated in triple-negative BC tissues, and its higher level was associated with much advanced clinical stage and poorer outcomes [8]. Moreover, circPTCH1 has been identified as a novel prognostic indicator and potential therapeutic target for patients with BC, as it promoted the malignant BC cell phenotype via autophagic level regulation [9]. However, the roles and mechanisms of circRNAs in BC remained unclear.

The present study performed high-throughput circRNA microarray sequencing and identified a set of distinct circRNAs in individuals with BC. A novel circRNA circRRM2 (hsa_circ_0052582) was identified, which originated from exons 6, 7, 8, and 9 of the RRM2 gene, and circRRM2 showed the significant upregulation in BC cell lines and tissues. We observed that circRRM2 promoted BC cell migration and invasion through functional gain and loss experiments. Additionally, a targeted regulatory correlation was verified among circRRM2, miR-27b-3p/miR-31-5p, and insulin-like growth factor 2 mRNA-binding protein 1 (IGF2BP1) through bioinformatic analysis and cellular experiments. Moreover, IGF2BP1 promoted BC cell migration and interacted with myelocytomatosis viral oncogene homolog (MYC), which functioned as the transcription factor of circRRM2. These findings demonstrated that circRRM2/IGF2BP1/MYC formed a positive feedback loop and facilitated the invasion behavior of cancer cells, which suggested the potential target for BC treatment.

## Materials and methods

### Patients and samples

Fourteen pairs of human BC and noncancerous tissues were collected from 2017 to 2021 at The Fourth Hospital of Hebei Medical University. Snap-frozen specimens were immediately collected after surgical removal and stored at − 80 °C until they were extracted. Seven of them were for circRNA expression profile analysis. The Fourth Hospital of Hebei Medical University’s Ethical Committee approved this study (approval ID: 2019MEC067) and all patients provided written informed consent.

### CircRNA microarray and computational analysis

CircRNA expression profile of seven pairs of human BC tissues and noncancerous tissues were analyzed using Arraystar Human circRNAs arrays (ArrayStar, Rockville, MD, USA), which contains 5396 probes specific to back-splicing junction regions of circRNA in humans. The circRNA microarray analysis was conducted by Sangon (Shanghai, China). The data were quantile normalized and processed using the R package. We used threshold values of ≥ 2 (or ≤  −2) fold change and an adjusted *P*-value of < 0.05. We obtained the circRNA dataset GSE111504 of BC from the GEO database to validate the expression level of circRNAs. The criteria for gene screening were |Log FC|> 2 and an adjusted *P*-value of < 0.05. The principal component analysis (PCA) was conducted to determine circRNAs with the ability to distinguish BC from non-BC (NT) tissue samples using the R package FactoMine [10, 11].

### Bioinformatics analysis

The potential biological roles of each parental gene of differentially expressed circRNA in BC were explored based on gene ontology (GO database, http://www.geneontology.org.) and Kyoto Encyclopedia of Genes and Genomes (KEGG database, http://www.genome.jp/kegg/) path enrichment analysis. A false discovery rate of < 0.05 was considered significant.

We obtained the sequence of hsa_circ_0052582, hsa_circ_0058113, hsa_circ_0058148, and hsa_circ_0060551 from circBase (www.circbase.org). Five online analysis tools were utilized to predict the potential miRNA candidates that might bind to the four circRNAs, including the CircBank database (http://www.circbank.cn/) [12], miRanda (http://www.microrna.org/), TargetScan (https://www.targetscan.org/vert_72/) [13], CircInteractome (https://omictools.com/circinteractome-tool) [14] and the cancer-specific circRNA database (http://geneyun.net/CSCD2) [15]. The downstream target genes of top miRNAs were predicted based on TargetScan, miRanda, and miRWalk (http://www.umm.uni-heidelberg.de/apps/zmf/mirwalk/) [16]. Cytoscape v3.5.1. (http://cytoscape.org/) was used to construct and visualize the circRNA-miRNA-mRNA network [17].

The single sample GSEA (ssGSEA) was performed to analyze the infiltration for 24 types of immunocytes in BC between IGF2BP1-high and IGF2BP1-low expression groups, using R package GSVA (http://www.bioconductor.org/packages/release/bioc/html/GSVA.html) [18, 19]. The correlation analysis between IGF2BP1 status of copy number variation and immunocytes infiltration in patients with BC was conducted using the TIMER platform (https://cistrome.shinyapps.io/timer/) [20]. The log-rank test was performed to analyze the prognostic significance of tumor-infiltrating immune cells using TIMER. Additionally, the specific correlation of IGF2BP1 with key immune checkpoints was also investigated using the Cancer Genome Atlas Breast Invasive Carcinoma (TCGA-BRCA) dataset.

### Cell culture

MCF-10A, the normal human mammary epithelial cells, and the BC cell lines (BT-474, BT-549, MCF-7, MDA-MB-231, MDA-MB-453, and T47D) were purchased from Procell (Wuhan, China). Cells were cultured under conditions recommended by the Procell and incubated at 37 °C and 5% CO_2_ in the incubator.

### RNA extraction and quantitative reverse transcription-polymerase chain reaction (qRT-PCR)

Total RNA was isolated from BC tissue and cells using TRIzol (Life Technologies, Carlsbad, CA, USA) following the manufacturer’s instructions. RNA was reverse transcripted using TransScript SuperMix (AT311-02, TransGen, Beijing, China). qRT-PCR was performed using TransStart Top Green qPCR SuperMix (TransGen, Beijing, China). The levels of candidate circRNAs or mRNAs were normalized by the β-actin expression. The miRNA level was normalized by the small nuclear U6 expression. All values were standardized with the 2^−ΔΔCT^ method. The sequences of oligonucleotides were listed in Additional file 1: Table S1.

### Western blotting

The cells were lysed using RIPA lysates (Solarbio, Beijing, China) with protease inhibitor (Beyotime, Beijing, China). Total proteins in the lysates were separated by 10% SDS–polyacrylamide gel electrophoresis and dotted to a polyvinylidene difluoride membrane (Millipore, MA, USA). We incubated the membranes with 5% skim milk powder for 1 h to block the non-specific binding at room temperature (21–25℃). Then the membrane was incubated overnight with the primary antibody. anti-IGF2BP1, anti-c-Myc, and anti-β-actin were purchased from Proteintech (Wuhan, China).

### RNA interference and plasmid transfection

SiRNAs targeting the back-spliced junction of circRRM2, and siRNAs targeting the mRNA of IGF2BP1 and MYC were synthesized by GenePharma (GenePharma Corporation, Shanghai, China). The overexpression plasmid of circRRM2 was constructed by inserting circRRM2 sequences into the pcDNA 3.1vector (Invitrogen, Carlsbad, CA, USA), named pcDNA-circRRM2 and the empty pcDNA 3.1 plasmids as a negative control [21]. The siRNAs or plasmids were transfected into BC cells using Lipofectamine 2000 (Invitrogen, Carlsbad, CA, USA). The siRNAs sequences were shown in Additional file 1: Table S2.

### FISH-immunofluorescence microscopy

We executed the FISH assay to visualize the circRRM2 and miR-27b-3p location in BT-549 cells. The hybridization was performed overnight with has_circ_0052582 and hsa-miR-27b-3p probes. The fluorescence microscope (Leica, Wetzlar, Germany) was used to photograph the slides. The circRRM2 probe for FISH was 5ʹ- CAT TGA AGA GAA ATT CCC TTG CTA AAA CCC -3ʹ and the hsa-miR-27b-3p probe for FISH was 5ʹ- GCA GAA CUU AGC CAC UGU GGC AGA AC TTA GCC ACT GTG -3ʹ.

### Wound healing assay

A cell scratch was made with a 200-μL pipette tip in each well 24 h after transfection. We measured the movement of cells in a scratch to assess cell motility. The healing rate (%) of cells was calculated with the formula: (0 h scratch width − 48 h scratch width)/0 h scratch width × 100%. The assays were performed in triplicate.

### Transwell assays

The 8-μm pore transwell chambers (Costar, MA, USA) were used to perform the transwell migration and invasion assays, either without (for migration assays) or with Matrigel (for invasion assays). We suspended 1.2 × 10^5^/mL infected cells in the upper chambers with a 200-μL serum-free medium and added a 500-μL complete medium into the bottom chambers. After a 24-h incubation, we removed the cells on the upper chambers, fixed the cells on the lower compartment with 4% paraformaldehyde, and imaged them with a microscope (Olympus, Tokyo, Japan).

### RNA immunoprecipitation (RIP)

We performed the RIP assays with the Dynabeads™ Protein G Immunoprecipitation Kit (DMEM, Invitrogen, CA, USA). RIP assay antibodies against AGO2 and IgG were purchased from Proteintech (Wuhan, China). The abundance of circRRM2 levels was measured by qRT-PCR assay.

### Co-immunoprecipitation (Co-IP)

Cells were lysed in NP40 lysis buffer with a protease inhibitor, RNase inhibitors, and PMSF (Solarbio, Beijing, China). Then, we centrifuged the lysates for 15 min at 14,000 × g and collected the supernatants. The supernatant samples were incubated with IGF2BP1 or IgG antibodies, combined with Protein A/G magnetic beads (Thermo Scientific, MA, USA) at 4 °C overnight with gentle shaking. Immunoprecipitates were eluted by boiling with immunoprecipitation-HA buffer and subjected to immunoblot analyses.

### Statistical analysis

Statistical analysis was conducted using GraphPad Prism 9.0 (GraphPad Software, CA, USA). Results were presented using the mean ± standard error of the mean. We evaluated the difference using the Student’s t-test, analysis of variance, or chi-square following the characteristics of data. The Kaplan–Meier analyses were used to perform the survival analysis. We performed the receiver operating characteristic (ROC) curve analysis using the pROC package in lung adenocarcinoma and healthy cases to assess the discrimination ability of the target gene. We included the potential parameters and constructed the nomogram with the rms package in R. The nomogram was a prognostic risk scoring system for BC, which could predict the survival probability of patients with BC for 1, 3, and 5 years. Differences were considered statistically significant at *P* < 0.05.

## Results

### Analysis of circRNA microarray profiles in BC

**Fig. 1:**
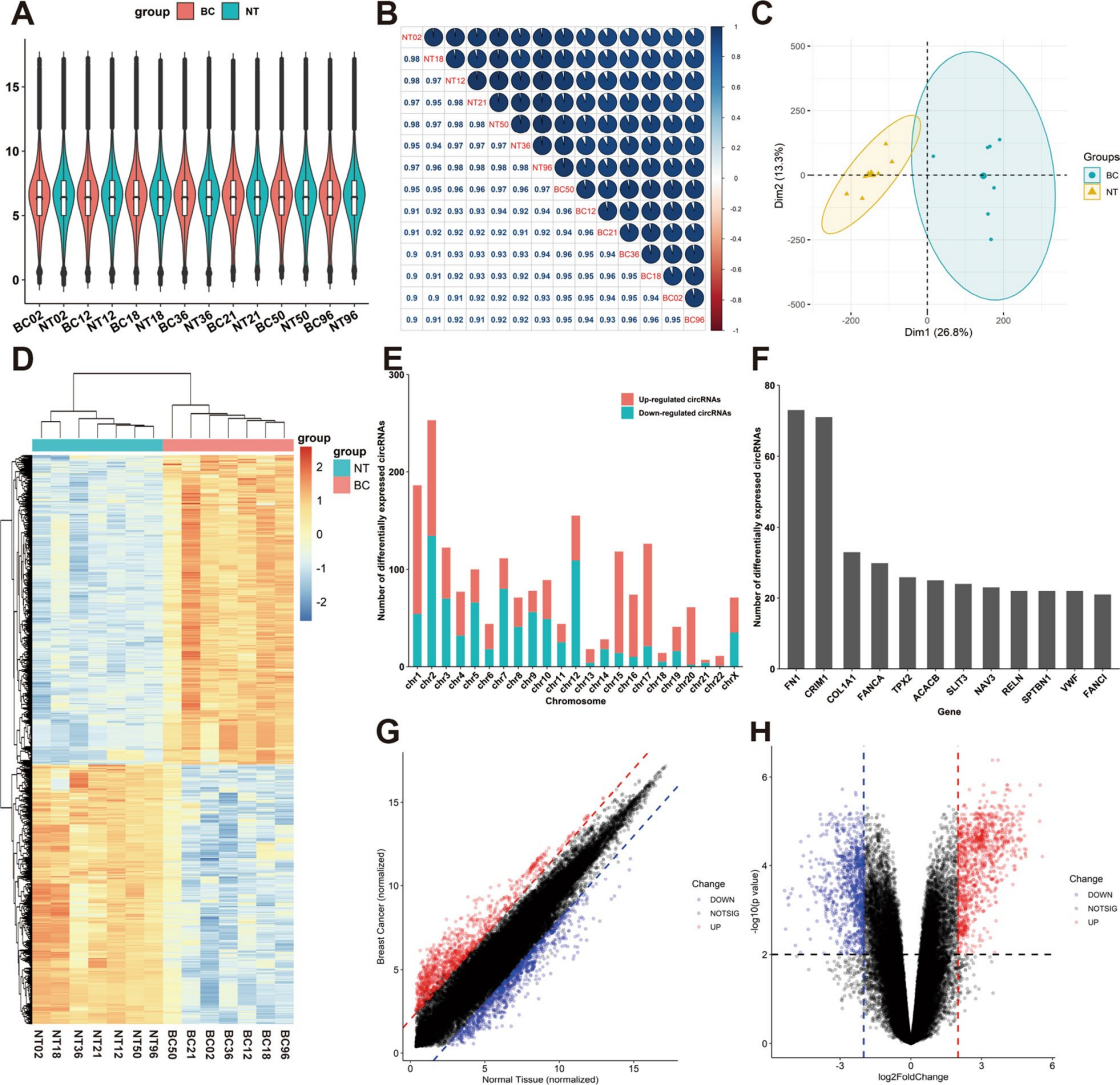
Analysis of circRNA microarray profiles in BC. **A** the consistency of BC-related circRNA expression among BC sample duplicates. **B** The circRNA expression correlation among BC sample duplicates. **C** The PCA analysis between paracancerous specimens and BC specimens. **D** The heatmap of differential expression of circRNA. **E** The location of the parent gene of upregulated and downregulated circRNA. **F** The top parent genes of differentially expressed circRNA. **G** The correlations between upregulated and downregulated circRNA. **H** The distribution of the upregulated and downregulated circRNA

We collected seven BC specimens and paired-paracancerous specimens from untreated patients with BC. CircRNA microarray was performed to reveal the differences in circRNA levels between the two groups, which showed the consistency of BC-related circRNA expression among sample duplicates (Fig. 1A). Pearson correlation indicated circRNA expression between BC samples had statistically correlated patterns (Fig. 1B). According to the PCA method, the samples were split into two groups in the two-dimensional coordinate system, and no outlier samples were detected (Fig. 1C). The screening thresholds for circRNA were adjusted to *P*-values of < 0.05 and |logFC| of > 2. A total of 1,911 dysregulated circRNA candidates were identified in the circRNA microarray, including 1040 upregulated and 871 downregulated circRNAs in BC. The dysregulated circRNA from BC was presented in the heatmap (Fig. 1D) and volcano plots (Fig. 1G, H). The majority of circRNAs were located in chromosomes 1 and 2 (Fig. 1E), and the top parental genes were FN1, CRIM, COL1A, and FANCA (Fig. 1F).

Subsequently, we identified four upregulated circRNAs as candidates (hsa_circ_0052582, hsa_circ_0058113, hsa_circ_0058148, and hsa_circ_0060551) according to the median expression value of > 7. The characteristic of their expression value in circRNA microarray was re-analyzed (Additional file 1: Fig. S1A, B), and their expression between BC samples had statistically correlated patterns (Additional file 1: Fig. S1C). The PCA results suggested that the above four circRNAs expressions could distinguish tumor samples from normal samples (Additional file 1: Fig. S1D).

GO enrichment and KEGG pathway analysis were performed using the 466 parent genes of dysregulated circRNAs that are identified in this study. Genes were mainly enriched in nuclear division and mitotic nuclear division in Biology Process (BP) (Additional file 1: Fig. S1E). Genes were chiefly associated with spindle and collagen-containing extracellular matrix in Cellular Component (CC) (Additional file 1: Fig. S1E). Genes mainly functioned in extracellular matrix structural constituent and microtubule-binding in Molecular Function (MF) (Additional file 1: Fig. S1E). The major KEGG pathways included cell cycle and oocyte meiosis (Additional file 1: Fig. S1F).

### The four candidate circRNAs are upregulated in BC

**Fig. 2:**
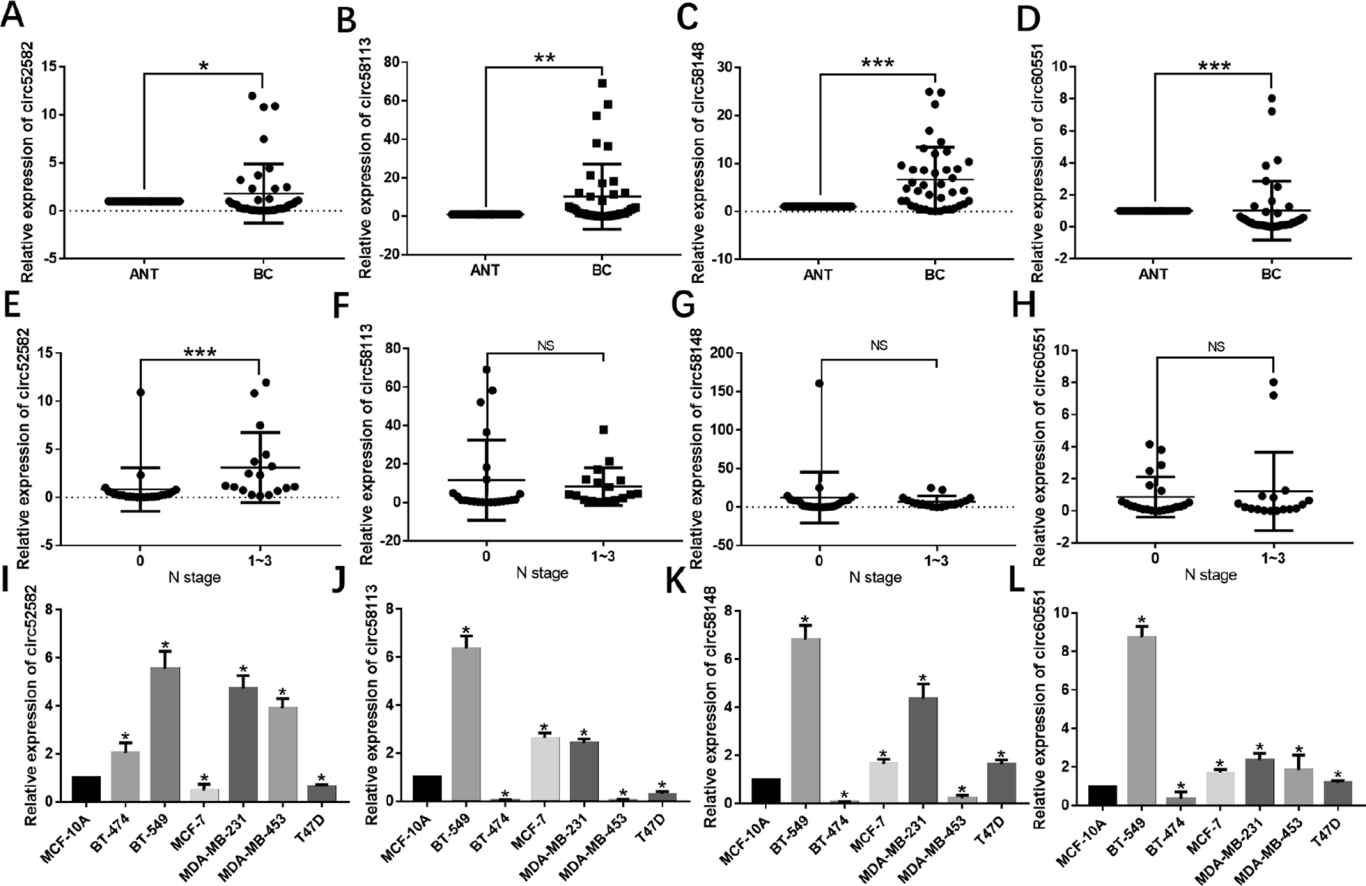
The four candidate circRNAs are upregulated in BC. Comparison of mRNA levels of hsa_circ_0052582 (**A**), hsa_circ_0058113 (**B**), hsa_circ_0058148 (**C**), and hsa_circ_0060551 (**D**) between BC and paired-paracancerous tissues. Comparison of levels of hsa_circ_0052582 (**E**), hsa_circ_0058113 (**F**), hsa_circ_0058148 (**G**), and hsa_circ_0060551 (**H**) in BC tissues classified by N stage (0 vs. 1–3). Comparison of hsa_circ_0052582 (**I**), hsa_circ_0058113 (**J**), hsa_circ_0058148 (**K**), and hsa_circ_0060551 (**L**) levels between BC cell lines (BT-474, BT-549, MCF-7, MDA-MB-231, MDA-MB-453, and T47D) and normal breast epithelial cells (MCF-10A). *P < 0.05, **P < 0.01, ***P < 0.001

We collected 40 BC tissues and 40 paired-paracancerous tissues from untreated patients with BC to validate the expression level of the four candidate circRNAs (hsa_circ_0052582, hsa_circ_0058113, hsa_circ_0058148, and hsa_circ_0060551). The mRNA level of the four candidates was determined using qRT-PCR assays. The levels of four candidates significantly increased in BC tissues as shown in Fig. 2A–E. Patients with a higher level of hsa_circ_0052582 expression had a more advanced N stage (*P* < 0.001, Fig. 2E), whereas the other three candidates had no relationship with N stages (Fig. 2F–H).

Subsequently, we further confirmed the level of the four candidates and obtained the circRNA expression data from the GEO dataset (GSE111504). The results indicated upregulated levels of the four candidates in BC tissues (Additional file 1: Fig. S2) and were positively associated with advanced T (Additional file 1: Fig. S3) and advanced N (Additional file 1: Fig. S4) stages. Meanwhile, we extracted the total RNA from BC cell lines (BT-474, BT-549, MCF-7, MDA-MB-231, MDA-MB-453, and T47D) and compared the expression levels of the four candidates with MCF-10A. The results of the qRT-PCR identified their high levels in multiple BC cell lines (Fig. 2I–L). In the following experiments, we selected BT-549 and MDA-MB-231 cells as the model cell lines for circRNAs’ higher expression levels.

### The characteristics of the four circRNA candidates

**Fig. 3:**
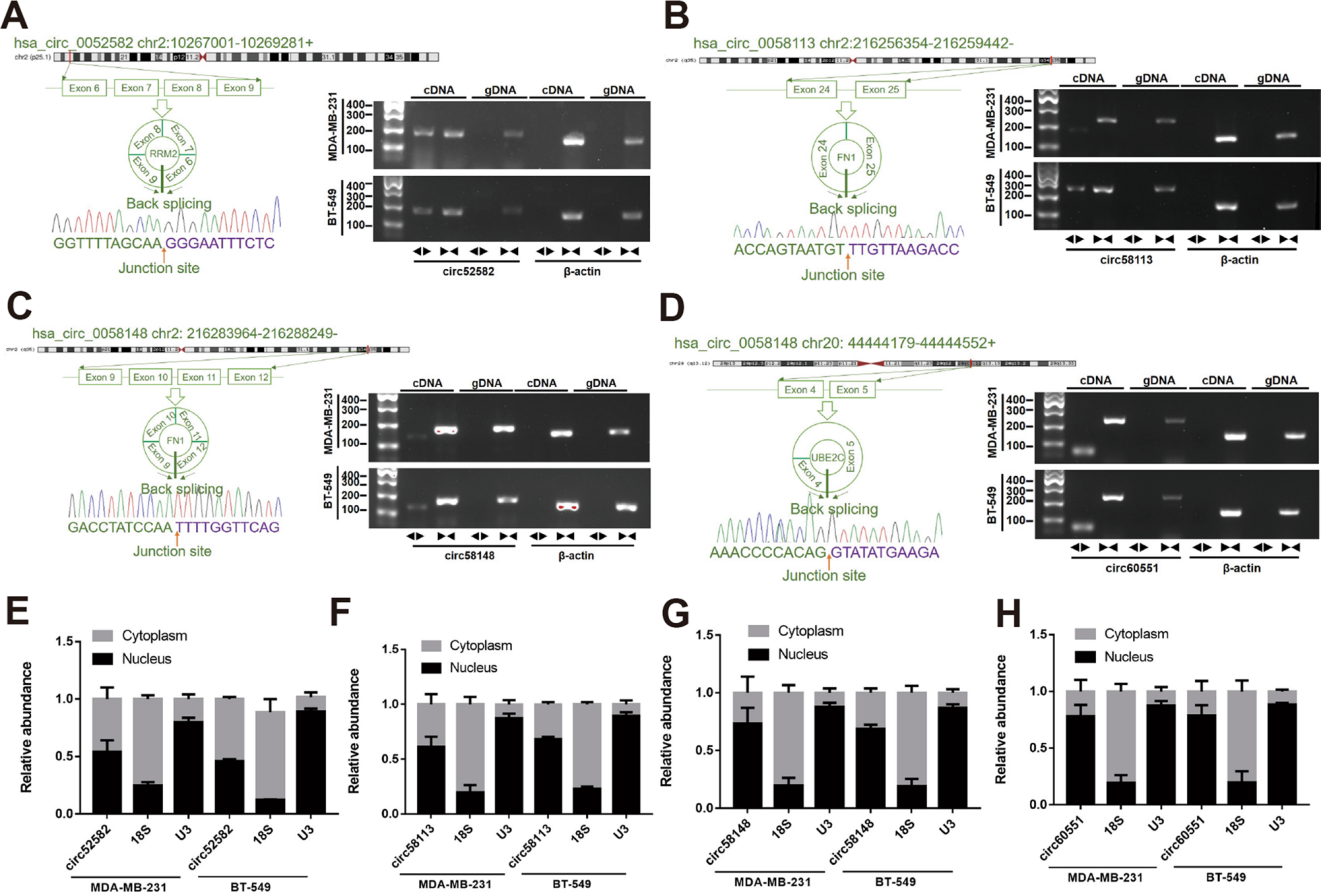
The characteristics of the four candidate circRNAs. **A**–**D** Schematic representation of the genomic localization of the four candidate circRNAs, the junction sites of back-splicing were verified by Sanger sequencing and the electrophoretogram of their PCR products by convergent and divergent primers. **E**–**H** The cellular localization of the four circRNAs

A set of specific divergent and convergent primers were designed to validate the circular structures of these circRNAs, and both cDNA and genomic DNA (gDNA) were used as the templates to amplify each circRNA, respectively (Fig. 3A–D). Convergent primers could amplify the linear transcripts in both cDNA and gDNA, and divergent primers could amplify the circRNAs in cDNA but not in gDNA (Fig. 3A–D). The back-splicing junction site of the four circRNAs was verified by Sanger sequencing (Fig. 3A–D), which confirmed their presence. Additionally, we explored the cellular localization of the four circRNAs after isolating the nuclear and cytosolic fractions. Hsa_circ_0052582 and hsa_circ_0058113 were detected in both nucleus and the cytoplasm of cells according to the PCR results in BT-549 or MDA-MB-231 cells (Fig. 3E, F), while hsa_circ_0058148 and hsa_circ_0060551 were predominantly localized in the nucleus (Fig. 3G, H).

### Construction of circRNA-miRNA-mRNA ceRNA network

We constructed a circRNA-miRNA-mRNA network map to better comprehend the interactions between circRNAs and miRNA or mediated mRNA. Firstly, we used five online algorithms, including CircBank, miRanda, TargetScan v7.1, CircInteractome, and cancer-specific circRNA database, to predict the circRNA-bound miRNA. The intersection of the predictions on these five datasets contained 412 miRNAs, and nine hub-miRNAs were identified by further screening, according to their expression level and prognostic value. Subsequently, a total of 8,831 target genes of nine hub-miRNAs were predicted by TargetScan v7.1, miRanda, and miRWalk 2.0. We screened the target genes and determined 537 hub genes based on the expression level and prognostic value. Finally, the circRNA-miRNA-mRNA ceRNA network was constructed with Cytoscape software 3.5.1 (Additional file 1: Fig. S5).

### CircRRM2 promotes BC cell migration and invasion

**Fig. 4:**
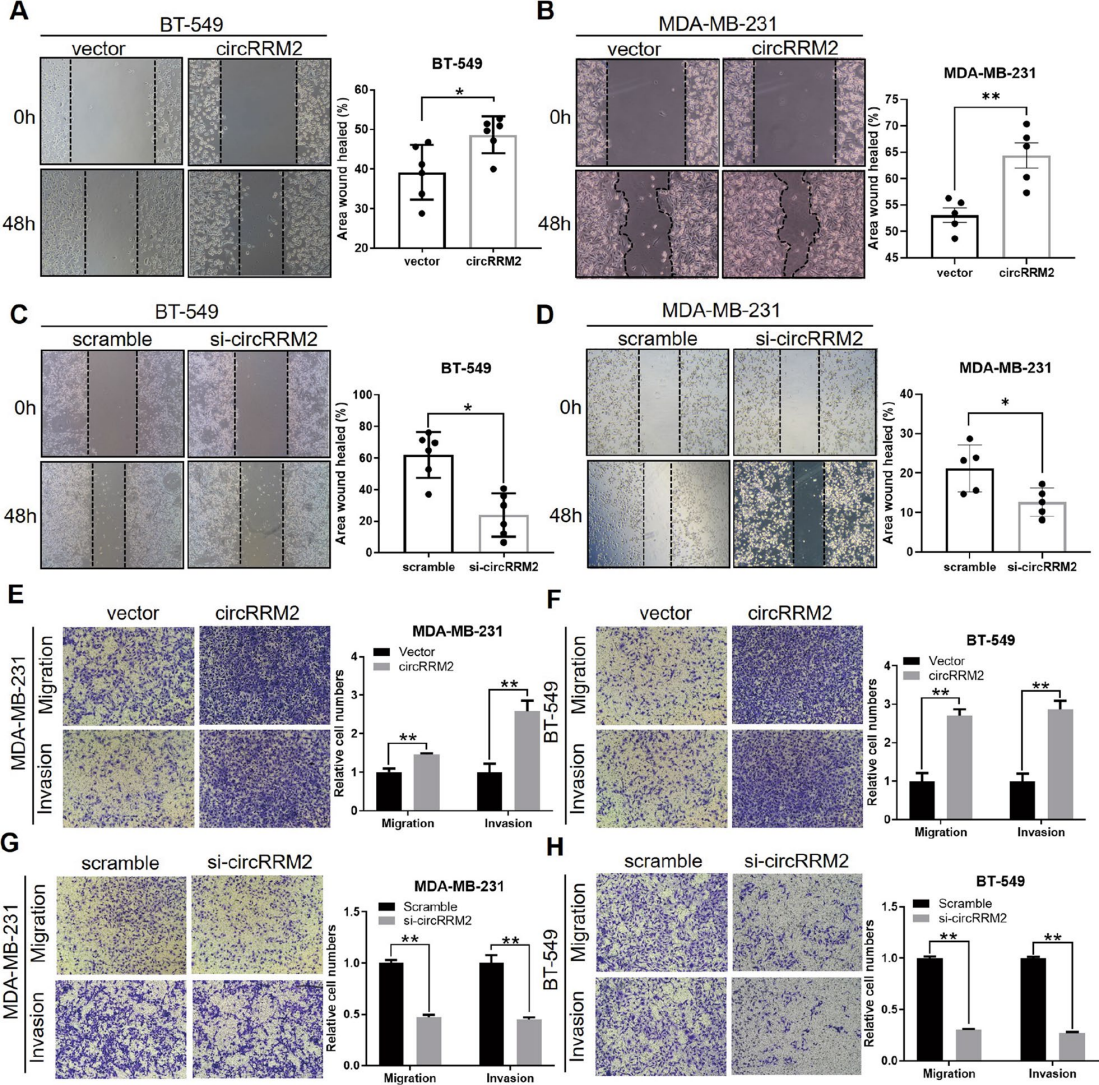
circRRM2 promotes BC cell migration and invasion. **A**–**D** Scratch wound healing assays of BC cells with circRRM2 overexpression or silencing. **E**–**H** Transwell assays of BC cell migration and invasion with circRRM2 overexpression or silencing. *P < 0.05, **P < 0.01

The scratch wound healing assay and transwell assay were utilized to investigate the functions of four circRNA candidates in BC cell migration. We designed the siRNA to target the back-splicing junction of circRNA (hsa_circ_0052582, hsa_circ_0058113, hsa_circ_0058148, and hsa_circ_0060551), respectively. After transfected with siRNA, scratch wound healing assays revealed that hsa_circ_0052582 silencing significantly suppressed cell migration (Fig. 4C, D). However, down-regulating the other three circRNAs (hsa_circ_0058113, hsa_circ_0058148, and hsa_circ_0060551) did not affect the cell migration ability. The parent gene of hsa_circ_0052582 is RRM2, thus we name it circRRM2, which is initially identified. The overexpression or knockdown of circRRM2 was success validated via qRT-PCR (Additional file 1: Figs. S6A and S6B). Additionally, the transwell assay illustrated that circRRM2 silencing significantly decreased BC cell migration and invasion (Fig. 4G, H). Then, pcDNA3.1-circRRM2 overexpression plasmid was constructed and transiently transfected into the BT-549 or MDA-MB-231 cells. Conversely, overexpressing circRRM2 remarkably promoted the migratory phenotype of BC cells (Fig. 4A, B, E, F).

### CircRRM2 serves as a miR-27b-3p/miR-31-5p sponge in BC cells

**Fig. 5:**
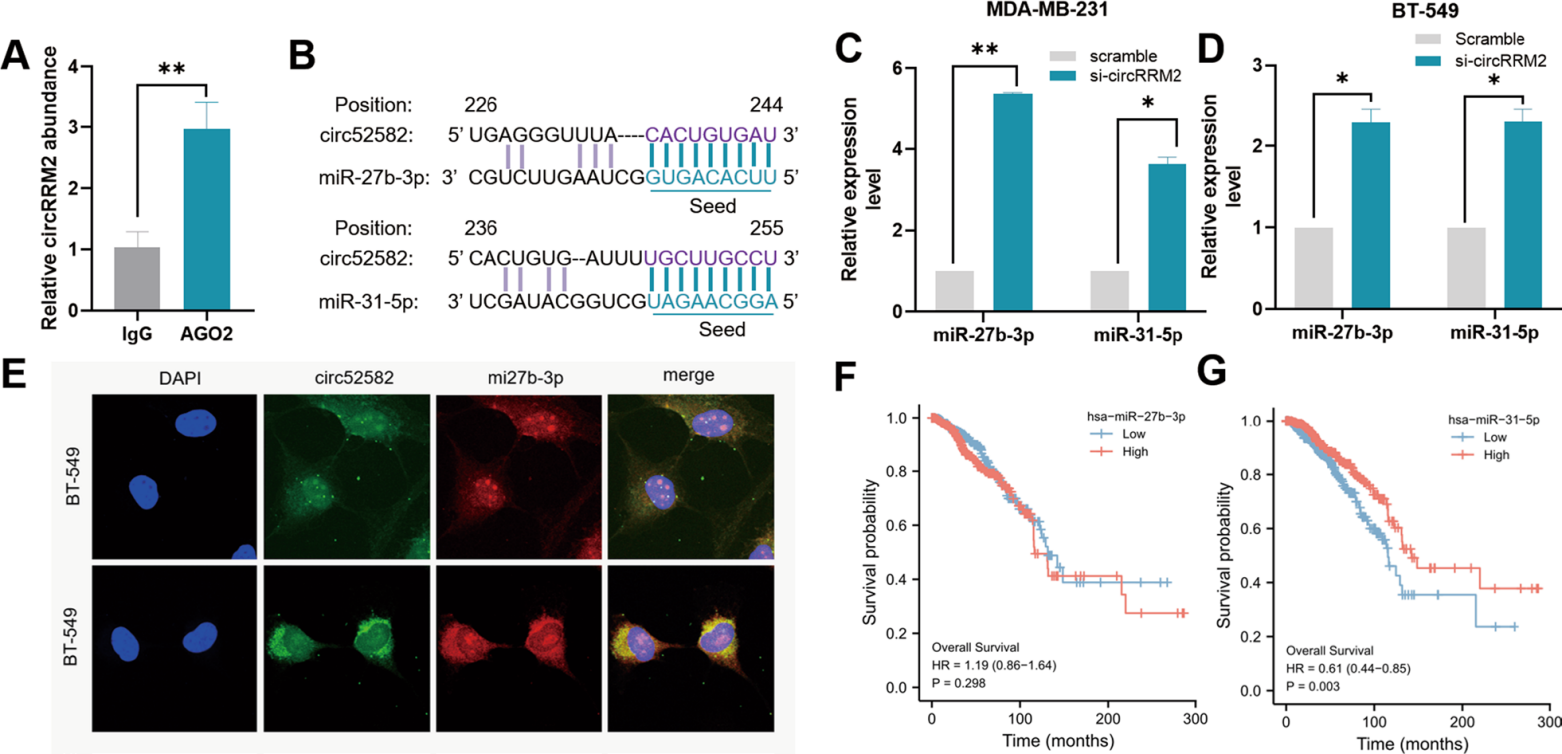
circRRM2 serves as a miR-27b-3p or miR-31-5p sponge in BC cells. **A** RIP assays using the AGO2 antibody for circRRM2 immunoprecipitation. **B** The seed sequences between circRRM2 and miR-27b-3p/miR-31-5p in the RNA22 database. qRT-PCR results of miR-27b-3p/miR-31-5p after circRRM2 knockdown in MDA-MB-231 (**C**) or BT-549 (**D**) cells. **E** The co-localization between circRRM2 and miR-27b-3p was observed in the cytoplasm using the FISH experiment. The prognostic value of miR-27b-3p (**F**)/miR-31-5p (**G**) in the TCGA-BRCA dataset was tested using the Kaplan–Meier analysis. *P < 0.05, **P < 0.01

For circRRM2 is localized in both the cytoplasm and nucleus (Fig. 3E), we hypothesized that it functioned through the ceRNA mechanism. AGO2 is the key component of the RNA-induced silencing complex (RISC) [22], and it could exert an important function in the ceRNA mechanism. Thus, we performed RIP experiments with an anti‐AGO2 antibody, and the enrichment of circRRM2 was observed (*P* < 0.01, Fig. 5A). The result revealed that circRRM2 could function via miRNA binding. Using the RNA22 database, circRRM2 was predicted to interact with miR-27b-3p or miR-31-5p, and the seed sequence was the longest with nine consecutive nucleotides (Fig. 5B). The mRNA level of miR-27b-3p or miR-31-5p was significantly upregulated in circRRM2-knockdown cells (Fig. 5C, D). The co-localization between circRRM2 and miR-27b-3p was observed in the cytoplasm using the FISH experiment (Fig. 5E). Next, we analyzed its correlation with overall survival using the TCGA-BRCA datasets to analyze the effect of miR-27b-3p or miR-31-5p expression on the prognosis of patients with BC. The KM curves indicated that the low miR-31-5p group was associated with poorer OS (*P* < 0.01, Fig. 5G), while miR-27b-3p did not associate with patient prognoses (*P* > 0.05, Fig. 5F).

### CircRRM2 facilitates cell migration via miR-27b-3p/miR-31-5p

**Fig. 6:**
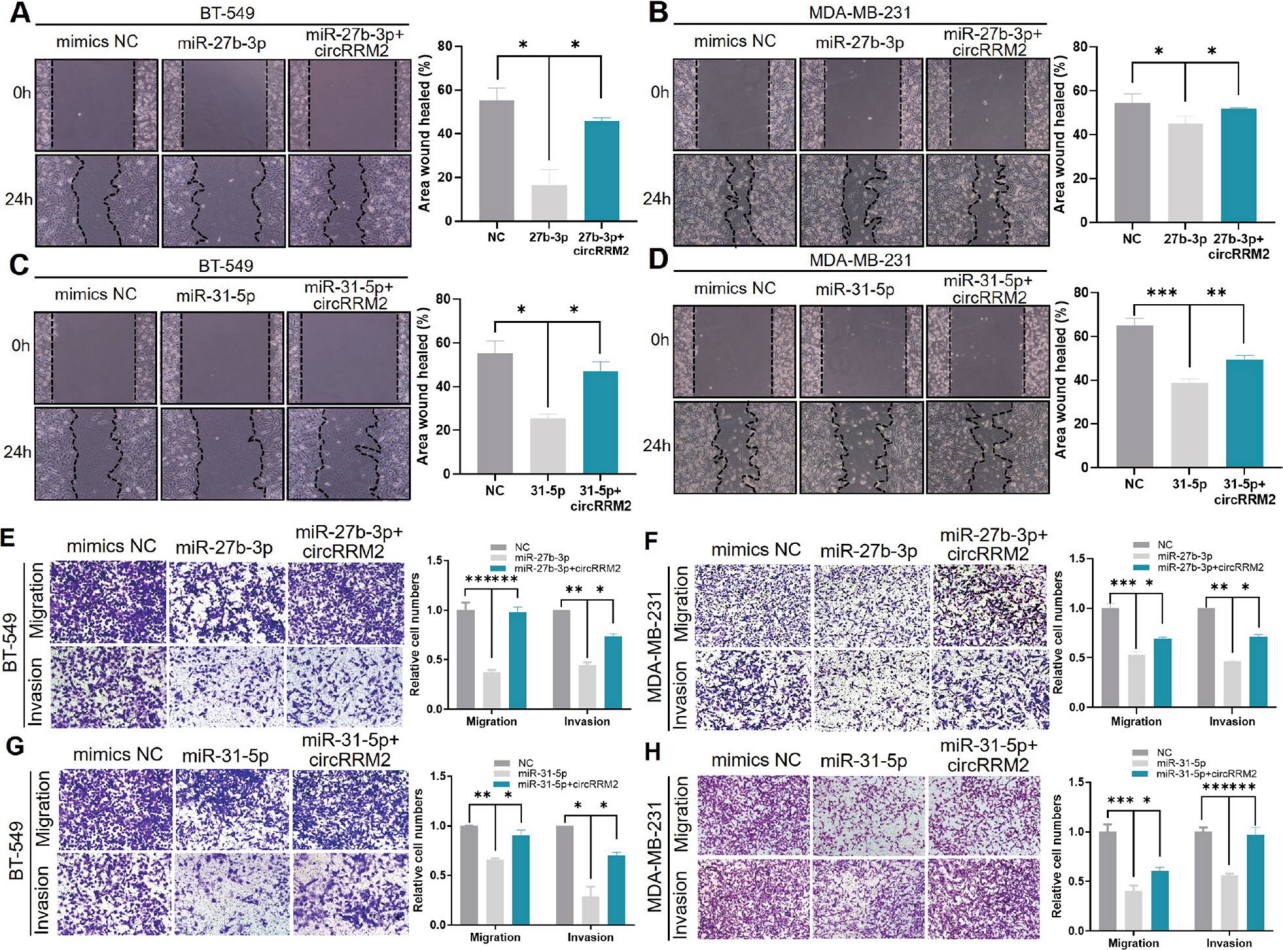
circRRM2 facilitates cell migration via miR-27b-3p/miR-31-5p. BT-549/MDA-MB-231 cells were transfected with miR-27b-3p (**A,B**) or miR-31-5p mimics (**C, D**), or combining with circRRM2 overexpression. The scratch wound healing assay was performed to determine the cell migration ability. The transwell assay was conducted to observe the cell migration/invasion when cells were transfected with miR-27b-3p (**E, F**) or miR-31-5p mimics (**G, H**) or combined with circRRM2 overexpression. *P < 0.05, **P < 0.01, ***P < 0.001

The scratch wound healing assay and transwell assay were utilized to explore the effects of miR-27b-3p or miR-31-5p on malignant BC cell phenotypes. Then, we transfected the miR-27b-3p or miR-31-5p mimics into BC cells, respectively. The results presented that miR-27b-3p overexpression inhibited both the BC cell migration (Fig. 6A, B, E, F) and the invasion (Fig. 6E, F) ability. Conversely, the migration phenotype was enhanced in the miR-27b-3p-inhibitor-transfected cells (Additional file 1: Fig. S6F). Consistently, upregulated miR-31-5p inhibited cell migration and invasion (Fig. 6C, D, G, H), while miR-31-5p knockdown promoted the cell migration (Additional file 1: Fig. S6G).

This study conducted rescue experiments to further elucidate the regulation of circRRM2 in BC cell migration and invasion through miR-27b-3p or miR-31-5p. We showed that circRRM2 overexpression rescued the migration of cells that were transfected with miR-27b-3p mimics using the scratch wound healing assay (Fig. 6A, B). Additionally, circRRM2 overexpression partially reverses malignant phenotypes of BC cells by transwell assays (Fig. 6C, D, G, H). Meanwhile, circRRM2 knockdown abolished the promotion of cell migration treated with miR-27b-3p inhibitor. Similar results were obtained when we simultaneously knocked down both circRRM2 and miR-31-5p in BT-549 cells (Additional file 1: Fig. S6G).

### CircRRM2 facilitates cell migration via regulating IGF2BP1

We identified 971 target genes that bind with both miR-27b-3p and miR-31-5p based on TargetScan v7.1, miRanda, and miRWalk 2.0 prediction results (Additional file 1: Fig. S7A). Then, we screened the target genes, which were upregulated and significant with OS of patients with BC, and obtained 23 target genes (Additional file 1: Figs. S7 and S8). High expression of eight genes (BRSK2, CACNA1B, CCNE1, EPN3, IGF2BP1, ONECUT2, PGLYRP4, and TMEM92) was associated with a poor OS prognosis (Additional file 1: Fig. S7B–I). Furthermore, we identified three genes (IGF2BP1, ONECUT2, and TMEM92) as candidates for their KM curves without crossing (Additional file 1: Fig. S7B–D).

**Fig. 7:**
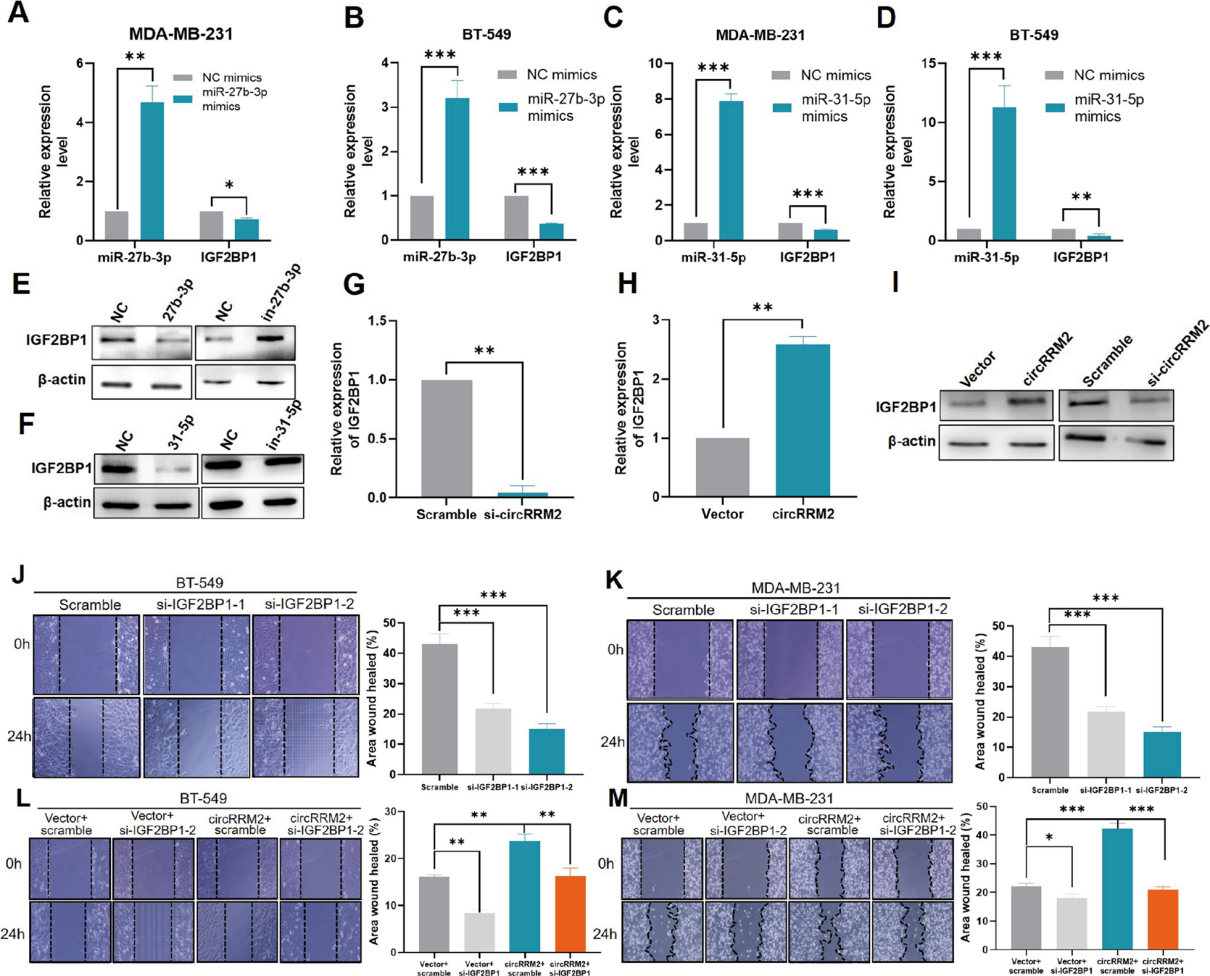
IGF2BP1 is the target gene of miR-27b-3p/miR-31-5p regulated by circRRM2. Alterations in the miR-27b-3p and IGF2BP levels in MDA-MB-231 (**A**) or BT-549 (**B**) cells following miR-27b-3p overexpression. Alterations in the miR-31-5p and IGF2BP1 levels in MDA-MB-231 (**C**) or BT-549 (**D**) cells following miR-31-5p overexpression. The IGF2BP1 protein level in the cells following the overexpression and knockdown of miR-27b-3p (**E**)/miR-31-5p (**F**) was examined using western blotting. Silencing or overexpression circRRM2, the expression of IGF2BP1 mRNA (**G**,** H**) and protein (**I**) was assessed by qRT-PCR and WB, respectively. Scratch wound healing assay showed the migration ability of IGF2BP1 silencing in BT-549 (**J**) or MDA-MB-231 (**K**) cells. The rescue effect of circRRM2 overexpression on IGF2BP1 knockdown in BT-549 (**L**) or MDA-MB-231 (**M**) cells was investigated. **P < 0.01, ***P < 0.001

The mRNA levels of IGF2BP1 were upregulated in BC cell lines, compared with that in MCF-10A (Additional file 1: Fig. S6C). The expression of IGF2BP1 was downregulated when miR-27b-3p in MDA-MB-231 or BT-549 cells was overexpressed (Fig. 7A, B). Similar results were obtained when cells were transfected with miR-31-5p mimics (Fig. 7C, D). WB also demonstrated similar results. The protein levels of IGF2BP1 represented the opposite trends with miR-27b-3p overexpressed or silencing (Fig. 7E), whereas the miR-31-5p mimics or inhibitor exerted no regulatory effect on IGF2BP1 at the protein level (Fig. 7F). Furthermore, circRRM2 knockdown in MDA-MB-231 cells resulted in IGF2BP1 expression downregulation at the (Fig. 7G, H) mRNA and (Fig. 7I) protein levels. These data suggested that circRRM2 exerted its ceRNA function by targeting IGF2BP1. Treatment with siRNA-1 and siRNA-2 of IGF2BP1 reduced the BT-549 (Fig. 7J) and MDA-MB-231 cell migratory ability (Fig. 7K), compared with that in scramble groups. Additionally, the rescue experiment was executed to study the circRRM2 regulation of the BC cell migration via IGF2BP1. Results demonstrated that circRRM2 knockdown can partially reverse the facilitation effect of overexpressed IGF2BP1 on BC cell migration (Fig. 7L, M). We simultaneously knocked down IGF2BP1 and overexpressed circRRM2 in BC cells in the transwell assay, and the cell invasive ability was partially rescued (Additional file 1: Fig. S9A, B).

### Upregulated IGF2BP1 promotes BC progression

**Fig. 8:**
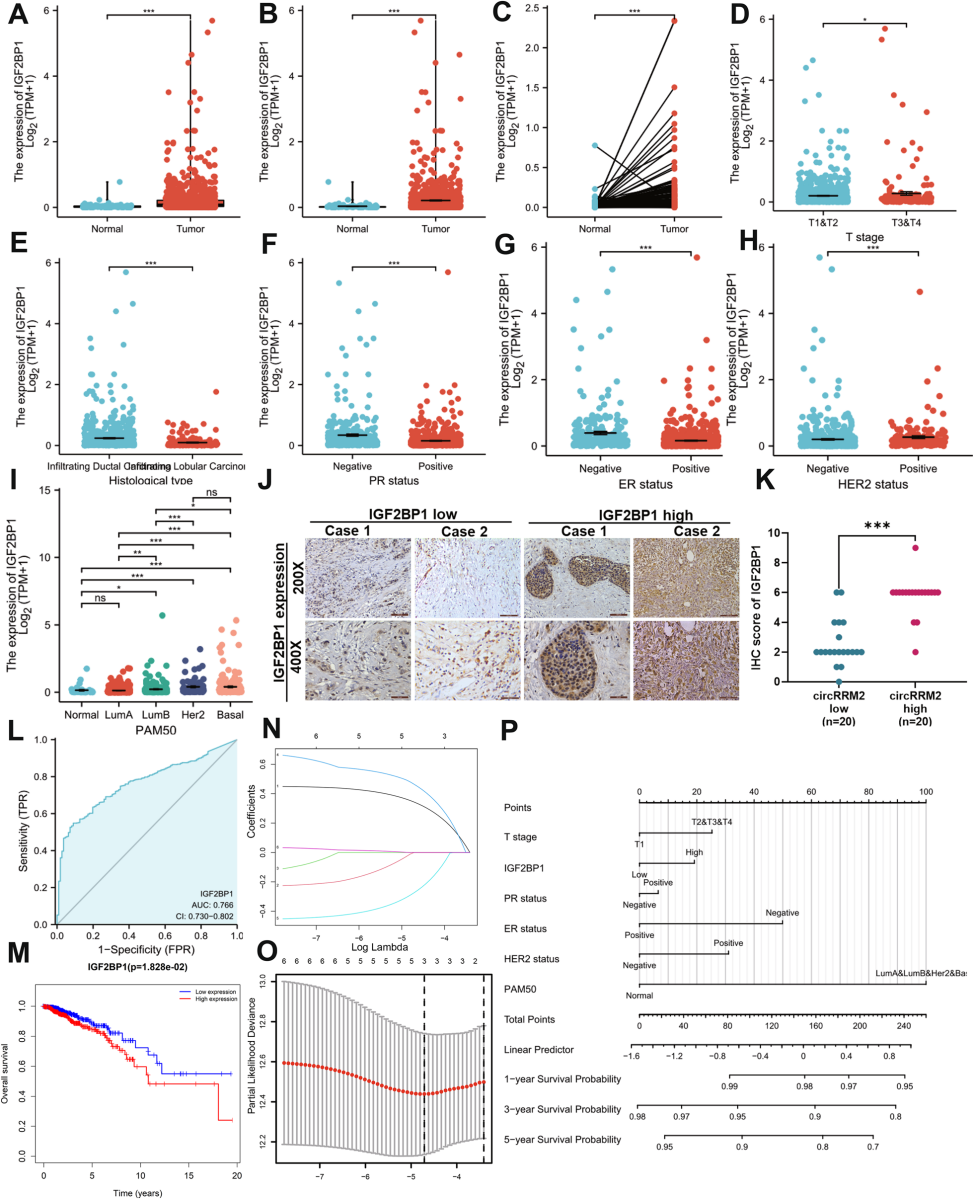
Analysis of the clinical significance of IGF2BP1. **A** Comparison of IGF2BP1 expression in BC (n = 1109) and normal samples collected by TCGA databases (n = 113). **B** Comparison of IGF2BP1 in BC (n = 1109) and normal samples collected by TCGA (n = 113) and GTEX databases (n = 179). **C** Comparison of IGF2BP1 in BC (n = 112) and paired normal samples (n = 112) collected by TCGA databases. **D** The differential expression of IGF2BP1 in the T1/T2 group (n = 906) and T3/T4 group (n = 174) was collected by TCGA databases. **E** The differential expression of IGF2BP1 in infiltrating ductal carcinoma (n = 772) and infiltrating lobular carcinoma (n = 205) was collected by TCGA databases. Analysis of IGF2BP1 expression level in BC tissues with different PR (**F**), ER (**G**), and HER2 status (**H**). **I** The IGF2BP1 expression among PAM50 subtypes. **J** Representative IHC pictures of IGF2BP1 in BC tissues. **K** Comparison of IHC scores of IGF2BP1 between the circRRM2-low (n = 20) and circRRM2-high groups (n = 20) in patients with BC. **L** ROC curves based on IGF2BP1 level to discriminate between normal and cancer in patients with BC. **M** The KM curve for the OS in patients with BC with high vs. low IGF2BP1 expression levels. **N** LASSO regression analysis for screening coefficients in predicting the OS for patients with BC. **O** The plot of trajectories of variables for predicting the probability of the OS for patients with BC. **P** Nomogram for predicting the 1-, 3‐, or 5‐year OS time in patients. *P < 0.05, **P < 0.01, ***P < 0.001

The TCGA-BRCA dataset was downloaded to explore the clinical significance of IGF2BP1 mRNA expression. IGF2BP1 was demonstrated as significantly increased in BC tissues (n = 1109) compared to that of normal tissues (n = 113) (*P* < 0.001, Fig. 8A), and its level was also higher compared with normal breast samples (n = 179) from the GTEX databases (*P* < 0.001, Fig. 8B). We identified that IGF2BP1 expression was significantly upregulated in BC tissues using the TCGA paired sample dataset (n = 112) (*P* < 0.001, Fig. 8C). The IGF2BP1 expression was significantly upregulated in patients with BC at T3/T4 stage compared with those at the T1/T2 stage (*P* < 0.001, Fig. 8D), and its level was much higher in ductal invasive tissues (*P* < 0.001; Fig. 8E). The tumor samples of PR-negative (*P* < 0.001, Fig. 8F), ER-negative (*P* < 0.001, Fig. 8G), or HER2-positive (*P* < 0.001, Fig. 8H) also possessed higher IGF2BP1 levels. We also compared the IGF2BP1 expression among PAM50 subtypes and found that its level was upregulated in all the subtypes, especially the basal group (Fig. 8I). Importantly, we validated the IGF2BP1 expression level using the IHC assay in 40 patients with BC, and IGF2BP1 represented a higher expression level in the high-circRRM2 group (Fig. 8J, K).

The ROC curve based on the IGF2BP1 level showed moderate accuracy to discriminate the outcome between normal and cancer (area under the curve = 0.766, confidence interval = 0.730–0.802, Fig. 8L). Survival curves indicated that patients with higher IGF2BP1 had a worse OS (*P* = 0.029, Fig. 8M) compared with the low IGF2BP1 group. We performed the LASSO regression analysis to screen the coefficients related to the OS of patients with BC (Fig. 8N), and the trajectories of variables were plotted in Fig. 8O. Finally, the significant variables, including IGF2BP1 level, T stage, PR status, ER status, HER2 status, and PAM50 subtype, were entered as candidates in the nomogram. The risk score calculated by the nomogram could predict the probability of OS for patients with BC. The calibration curve was also plotted to measure the performance of the nomogram, which showed moderate predictive power (C-index = 0.669, Additional file 1: Fig. S10).

### Analysis of immune cell infiltration related to IGF2BP1 in patients with BC

We performed the ssGSEA to explore the infiltration of 24 types of immunocytes in BC between the IGF2BP1-high and IGF2BP1-low expression groups. The result demonstrated that IGF2BP1 expression presented a variety of correlations to immune cell infiltration (Additional file 1: Fig. S11A). IGF2BP1 high level was positively associated with the infiltration of macrophages, Th1 cells, and Th2 cells, and negatively to NK CD56 bright cells, eosinophilia, and mast cells (Additional file 1: Fig. S11A). We further found that IGF2BP1 expression positively correlated with the infiltration of T cells, B cells, and macrophages in patients with BC using the TIMER platform (Additional file 1: Fig. S11C). Additionally, higher B cell infiltration levels predicted a better prognosis in OS in patients with BC (Additional file 1: Fig. S11D). Based on copy number variation of IGF2BP1 status using TIMER, we divided the patients with BC into five groups, including arm-level deletion, deep deletion, diploid/normal, high amplification, and arm-level gain. We identified a significant downregulation in the levels of B cells, CD4 + T cells, CD8 + T cells, neutrophils, macrophages, and dendritic cells in the arm-level deletion group (Additional file 1: Fig. S11E). Moreover, the correlation between the IGF2BP1 expression level and checkpoint-related genes was observed (Additional file 1: Fig. S11B). Patients with BC with high IGF2BP1 expression had significantly higher levels of CD274, CTLA4, HAVCR2, LAG3, PDCD1, PDCD1LG2, and TIGIT (Additional file 1: Fig. S11B).

### Mutational analysis of IGF2BP1

We analyzed the characteristic of missense mutation and PTM sites located in IGF2BP1 in BC samples using the cBioPortal database (Additional file 1: Fig. S12). The results exhibited that amplification was the predominant mutation type, and covered 6% of the entire protein length (Additional file 1: Fig. S12A). The TMB score was 264, and the MSI mantis score ranged from 0.22 to 0.83 (Additional file 1: Fig. S12A). The high-frequency mutation site was A240T located at the KH-1 domain of IGF2BP1, and multiple phosphorylation and acetylation sites were prevalent in the IGF2BP1 amino acid sequence (Additional file 1: Fig. S12B).

### The mechanism of the positive feedback loop of circRRM2/IGF2BP1/MYC

**Fig. 9:**
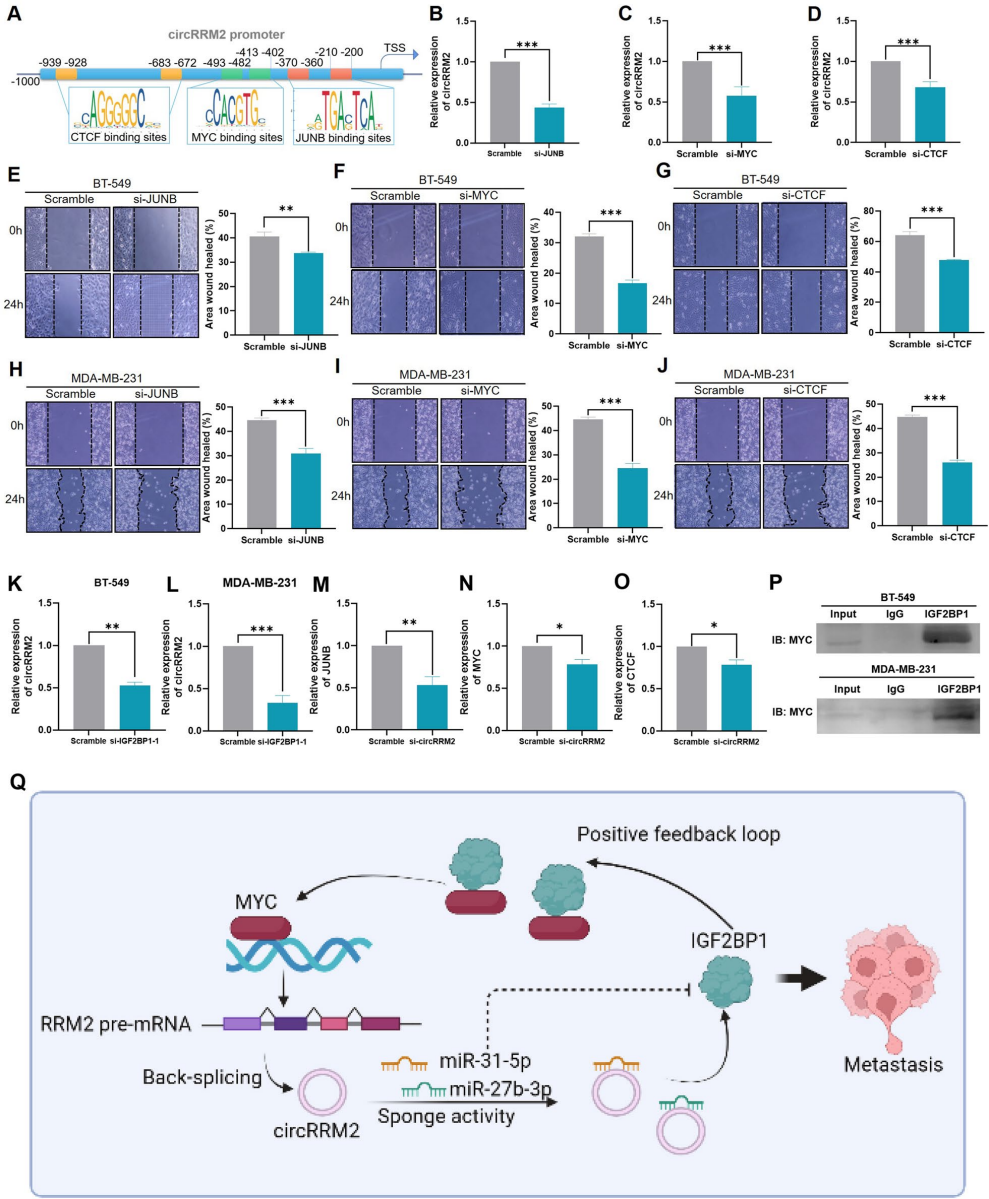
Validation of the MYC function as a transcription factor of circRRM2 and a positive feedback loop of circRRM2/IGF2BP1/MYC. **A** Schematic of transcription factor binding sites of the circRRM2 promoter. qRT-PCR results of circRRM2 level in BT-549 cells after silencing JUNB (**B**), MYC (**C**), or CTCF (**D**). BC cells were tested to determine their migration ability in scratch wound healing assays after silencing JUNB (**E/H**), MYC (**F/I**), or CTCF (**G/J**). The relative expression of circRRM2 after silencing IGF2BP1 in BT-549 (**K**) or MDA-MB-231 (**L**) cells. The relative expression of JUNB (**M**), MYC (**N**), or CTCF (**O**) after silencing IGF2BP1 in BT-549 cells. **P** CoIP and WB experiments to detect the binding of MYC to IGF2BP1. **Q** Diagram illustrating the proposed mechanism of positive feedback loop of circRRM2/IGF2BP1/MYC. *P < 0.05, **P < 0.01, ***P < 0.001

We obtained a 1000 bp sequence upstream of the circRRM2 transcription start site to conduct further analysis to explore the upstream regulators of circRRM2. Then, the candidate transcriptional factors were identified as MYC, JUNB, and CTCF, using the JASPAR database (Fig. 9A). We observed that circRRM2 was downregulated after decreasing JUNB (Fig. 9B), MYC (Fig. 9C), or CTCF (Fig. 9D), respectively. Meanwhile, the migration ability of BC cells was significantly restrained, as the level of JUNB (Fig. 9E/H), MYC (Fig. 9F/I), or CTCF (Fig. 9G/J) was decreased. Interestingly, the circRRM2 level was also significantly reduced when the cells were transfected with IGF2BP1 siRNA (Fig. 9K, L). The MYC, JUNB, and CTCF levels were also reduced with concomitant IGF2BP1 expression decreases (Fig. 9M–O). The above results suggested that IGF2BP1 positively regulated the circRRM2 expression through interacting with MYC/JUNB/CTCF. Then, we noted that MYC was one of the candidates bound to IGF2BP1 predicted by BioGRID (Additional file 1: Fig. S13). Compared with matched paracancerous tissues, MYC mRNA expression was up‐regulated in BC tissues (Additional file 1: Fig. S6D). Moreover, we analyzed the expression correlation between circRRM2 and MYC in 25 BC tissues. The result indicated the positive expression correlation between circRRM2 and MYC in BC (R^2^ = 0.79, *P* < 0.0001, Additional file 1: Fig. S6E). CoIP and WB assays were performed to detect the binding of MYC to IGF2BP1. The IGF2BP1 antibody induced a significant enrichment of MYC in comparison to the IgG antibody (Fig. 9P). Our results illustrated that IGF2BP1 upregulated the transcriptional circRRM2 activation via interacting with MYC, which lead to an increased IGF2BP1 level (Fig. 9Q). Therefore, circRRM2/IGF2BP1/MYC formed a positive feedback loop and facilitated the invasion behavior of cancer cells.

## Discussion

This study elucidated a number of dysregulated circRNAs in BC compared with adjacent normal tissues [4]. Here we identified that circRRM2 was increased in BC tissues, and was capable of promoting BC cell migration and invasion. CircRRM2 acted as an oncogene by competing with miR-31-5p/miR-27b-3p to drive the IGF2BP1 expression. Rescue assays demonstrated that circRRM2 knockdown can partially reverse the facilitation effect of overexpressed IGF2BP1 on BC cell migration. Importantly, IGF2BP1 upregulated the circRRM2 level via interacting with MYC, which lead to an increased IGF2BP1 level. Therefore, circRRM2/IGF2BP1/MYC formed a positive feedback loop and facilitated the metastasis in patients with BC.

CircRNAs perform specific functions depending on their subcellular locations [6]. In the cytoplasm, circRNAs frequently exert their functions by sponging miRNAs [6]. This study revealed circRRM2 in both the cytoplasm and nucleus of BC cells (Fig. 3E). Ago2 is the key component of RISC involved in the ceRNA mechanism [22], and the anti‐Ago2 antibody was able to enrich circRRM2 by RIP assays (Fig. 5A). The above results further confirmed that circRRM2 could function via miRNA binding. Subsequently, we performed the bioinformatics analysis and found that circRRM2 might bind to miR-27b-3p or miR-31-5p (Fig. 5B). The mRNA level of miR-27b-3p or miR-31-5p significantly increased in circRRM2-knockdown cells (Fig. 5C, D). Additionally, we observed the co-localization between circRRM2 and miR-27b-3p in the cytoplasm using the FISH experiment (Fig. 5E). The KM curves indicated that the low miR-31-5p group was associated with the poorer OS using TCGA-BRCA datasets (Fig. 5G). A previous study showed a significantly reduced miR-31-5p in renal cell carcinoma (RCC) tissues and its downregulation correlated with poorer prognosis in patients with RCC [23]. The finding is consistent with our results. We subsequently assessed the invasive phenotype of miR-27b-3p or miR-31-5p transfecting its mimics or inhibitor into BC cells and identified that both miR-27b-3p and miR-31-5p exerted a tumor-suppressor on BC (Fig. 6 and Additional file 1: Fig. S6). This trend is consistent with those reported in previous studies [24–26]. Further rescue experiments revealed that circRRM2 significantly attenuated miR-27b-3p’ effects on BC cell invasive ability (Fig. 6 and Additional file 1: Fig. S6). These results suggested that circRRM2 may exert its biological functions through the ceRNA mechanism in BC.

Subsequently, we identified IGF2BP1 as the candidate for miR-31-5p/miR-27b-3p target gene for its upregulated expression associated with poor OS in patients with BC (Additional file 1: Fig. S7B). The IGF2BP1 mRNA levels were downregulated in BC cells when miR-27b-3p or miR-31-5p was overexpressed (Fig. 7A–D). The IGF2BP1 protein level was suppressed by the mimics of miR-27b-3p but not miR-31-5p (Fig. 7E, F), possibly because miR-27b-3p might involve in the post-transcriptional IGF2BP1 processing. IGF2BP1 belongs to the IGF2BP family, and its gene contains two RNA recognition motifs and four K-homology domains [27]. The protein binds to the mRNAs of certain genes, such as insulin-like growth factor 2 or beta-actin repeat-containing protein and regulates their translation [27]. In BC cells, IGF2BP1 is necessary for clonogenic activity and its function is served by either the full-length IGF2BP1 or the N-terminally truncated version [28]. Additionally, IGF2BP1 enhances the MIR210HG stability, which functions as an oncogenic lncRNA, and contributes to BC progression [29]. Our study revealed similar results that siRNA treatment of IGF2BP1 reduced the migratory ability of BC cells (Fig. 7J, K). We also executed the rescue experiment, and the results demonstrated that circRRM2 knockdown can partially reverse the facilitation effect of overexpressed IGF2BP1 on BC cell migration or invasion (Fig. 7L, M, Additional file 1: Fig. S9). These results suggested that circRRM2 may function as a ceRNA to regulate IGF2BP1 in BC.

Additionally, we illustrated the role of IGF2BP1 in BC clinical significance and infiltrating immune cells in the tumor microenvironment through bioinformatic analysis. BC tissues showed significant IGF2BP1 expression upregulation (Fig. 8A–C). The high IGF2BP1 level was positively associated with high T stage, PR-negative, ER-negative, or HER2-positive in patients with BC (Fig. 8D, F ,G). We also validated that the high- circRRM2 group more strongly expressed IGF2BP1 by IHC assay in 40 patients with BC (Fig. 8J, K). The ROC curve accuracy was 0.766, which indicated that the discriminatory ability for BC from the normal by the IGF2BP1 level was moderate (Fig. 8L). The Kaplan–Meier analysis revealed that patients with higher IGF2BP1 had a worse OS (Fig. 8M). The above results demonstrated that IGF2BP1 was an oncogene in BC. Previous studies identified that IGF2BP1 promoted tumor progression, which contributed to the malignant phenotypes of tumor cells, and multiple types of human cancers showed poor OS and metastasis when IGF2BP1 was upregulated [27, 30, 31]. Thus, our results further proved the above conclusions. Additionally, the IGF2BP1 expression is positively related to the infiltration of T cells, B cells, and macrophages, and is also associated with checkpoint-related genes in patients with BC (Additional file 1: Fig. S11A and C). IGF2BP1 could function as an m6A reader and enrich the 3'-UTR of immune checkpoint PD-L1 mRNA, thus enhancing the mRNA stability of PD-L1 in bladder cancer [32]. It might be the interpretation of our results.

The proto-oncogene MYC plays an essential role in the progression of the cell cycle, apoptosis, and cell transformation [33]. Complexes formed by MYC and MAX bind to DNA consensus sequences in the E box and regulate the transcription of specific genes [33]. MYC in cancer could bind to the VEGFA promoter, thereby stimulating the VEGFA production and subsequently sprouting angiogenesis [34, 35]. The present study identified that two MYC transcription factor binding sites were present in the circRRM2 promoter (Fig. 9A), and circRRM2 was downregulated after MYC in BC cells (Fig. 9B). Thus, we speculated that MYC may contribute to the circRRM2 transcriptional activation. Interestingly, we also found that MYC was one of the candidates bound to IGF2BP1 predicted by BioGRID (Additional file 1: Fig. S13), and the binding of MYC to IGF2BP1 was validated by CoIP and WB experiments (Fig. 9P). A previous study reported that IGF2BP1 could retain the stability of MYC mRNA in BC cells, by increasing the binding of IGF2BP1 with the m6A-modified MYC mRNA of the coding region instability determinant [36, 37]. This could explain the interaction mechanism between IGF2BP1 and MYC and the downstream effects; hence, further investigations are required in our study. Moreover, the circRRM2 level was significantly reduced when the cells were transfected with IGF2BP1 siRNA (Fig. 9K, L). The MYC levels were also reduced with IGF2BP1 level degression (Fig. 9N). The above results suggested that IGF2BP1 positively regulated the circRRM2 expression through interacting with MYC/JUNB/CTCF. Therefore, we hypothesized that circRRM2/IGF2BP1/MYC formed a positive feedback loop and promoted BC metastasis.

Our study has some limitations. First, the role of circRRM2 in promoting cancer metastasis was not validated with animal experiments. Second, the miRNA binding sites with circRRM2 or IGF2BP1 were predicted using the bioinformatics method, which needs to be experimentally validated. Third, the interaction of IGF2BP1 with MYC and circRRM2 level regulation in BC progression still needs further investigation; hence, our future studies will continue to focus on this issue.

## Conclusions

In summary, our results confirm that upregulated circRRM2 functions as oncogenic in BC metastasis. circRRM2 competitively binds miR-31-5p/miR-27b-3p to abolish the suppressive effect on IGF2BP1, then promotes BC cell migration and invasion. The results reveal that a feed-forward loop of circRRM2/IGF2BP1/MYC enforced the circRRM2 expression. Our study may offer a novel perspective on the molecular mechanism of BC metastasis and a potential target for BC treatment.

## Supplementary Information

**Additional file 1:** **Fig. S1.** Analysis of four candidate circRNAs and enrichment analysis of parent genes. **Fig. S2.** The mRNA levels of hsa_circ_0052582, hsa_circ_0058113, hsa_circ_0058148, and hsa_circ_0060551 in patients with BC from the GEO dataset (GSE111504). **Fig. S3.** The mRNA levels of hsa_circ_0052582, hsa_circ_0058113, hsa_circ_0058148, and hsa_circ_0060551 in patients with BC at different T stages from the GEO dataset (GSE111504). **Fig. S4.** The mRNA levels of hsa_circ_0052582, hsa_circ_0058113, hsa_circ_0058148, and hsa_circ_0060551 in patients with BC at different N stages from the GEO dataset (GSE111504). **Fig. S5.** CircRNA-miRNA-mRNA ceRNA network. **Fig. S6.** Expression level of circRRM2/IGF2BP1/MYC in BC and the migration phenotype of miR-27b-3p/miR-31-5p inhibitor in BC cells. Overexpression (A) or knockout (B) efficiency of circRRM2 in BT-549 and MDA-MB-231 was verified by RT qPCR. (C) The levels of IGF2BP1 in BC cells. (D) Expression level of MYC in BC tissues. (E) Correlation analysis of circRRM2 and MYC in BC tissues. (F-G) circRRM2 knockdown abolished the suppression of cell migration treated with miR-27b-3p/miR-31-5p inhibitor. BT-549 cells were transfected with miR-27b-3p (F) or miR-31-5p (G) inhibitor, and the scratch wound healing assay was performed to measure the ability of cell migration. The rescue assay was conducted by co-transfecting the circRRM2 plasmid. *P < 0.05, **P < 0.01, ***P < 0.001. **Fig. S7.** Prediction and prognostic value of target genes binding with both miR-27b-3p and miR-31-5p. **Fig. S8.** Prognostic value of target genes binding with both miR-27b-3p and miR-31-5p. *P < 0.05, **P < 0.01, ***P < 0.001. **Fig. S9.** The transwell assay in BC cell transfected with circRRM2 plasmid and IGF2BP1 siRNA. The transwell assay was performed to detect the rescue effect of overexpression of circRRM2 on IGF2BP1 knockdown in BT-549 (A) or MDA-MB-231 (B) cells. **Fig. S10.** Calibration plot of the nomogram to predict the probability of the OS in patients with BC at 1, 3, and 5 years. **Fig. S11. **Analysis of immune cell infiltration related to IGF2BP1 in patients with BC. (A) Lollipop plot shows the correlation between IGF2BP1 level and immunocytes infiltration. (B) Correlations between IGF2BP1 and immune checkpoint-related genes. (C) Scatter plot of correlation between IGF2BP1 and immunocytes infiltration. (D) KM curve of immunocytes infiltration status and OS in patients with BC. (E) Comparison of immunocytes infiltration related with copy number variation of IGF2BP1. *P < 0.05, **P < 0.01, ***P < 0.001. **Fig. S12.** Mutational analysis of IGF2BP1. (A) The oncoprint of IGF2BP1 was obtained using cBioPortal. (B) Analysis of PTM sites in IGF2BP1. **Fig. S13.** Network of candidates binding to IGF2BP1 constructed using the BioGRID database. **Table S1.** Primers used for qRT-PCR. **Table S2.** The sequences of siRNAs.

## Data Availability

All data in our study are available upon request.

## Abbreviations

BC: Breast cancer
BP: Biology process
CC: Cellular component
ceRNA: Competing endogenous RNA
circRNA: Circular RNA
co-IP: Co-immunoprecipitation
FISH: Fluorescence in situ hybridization
IGF2BP1: Insulin-like growth factor 2 mRNA-binding protein 1
MF: Molecular function
ncRNA: Non-coding RNA
PTM: Posttranslational modification
qRT-PCR: Quantitative reverse transcription-polymerase chain reaction
RIP: RNA immunoprecipitation
RISC: RNA-induced silencing complex
ROC: Receiver operating characteristic
TCGA-BRCA: The Cancer Genome Atlas Breast Invasive Carcinoma
