# Supplementary material for: A novel feedback regulated loop of circRRM2-IGF2BP1-MYC promotes breast cancer metastasis
Source: Cancer Cell Int. 2023 Mar 25;23:54. doi: 10.1186/s12935-023-02895-w (PMC10039515; doi:10.1186/s12935-023-02895-w)
Supplement: Supplementary file 1 — Additional file 1: Fig. S1. Analysis of four candidate circRNAs and enrichment analysis of parent genes. Fig. S2. The mRNA levels of hsa_circ_0052582, hsa_circ_0058113, hsa_circ_0058148, and hsa_circ_0060551 in patients with BC from the GEO dataset (GSE111504). Fig. S3. The mRNA levels of hsa_circ_0052582, hsa_circ_0058113, hsa_circ_0058148, and hsa_circ_0060551 in patients with BC at different T stages from the GEO dataset (GSE111504). Fig. S4. The mRNA levels of hsa_circ_0052582, hsa_circ_0058113, hsa_circ_0058148, and hsa_circ_0060551 in patients with BC at different N stages from the GEO dataset (GSE111504). Fig. S5. CircRNA-miRNA-mRNA ceRNA network. Fig. S6. Expression level of circRRM2/IGF2BP1/MYC in BC and the migration phenotype of miR-27b-3p/miR-31-5p inhibitor in BC cells. Overexpression (A) or knockout (B) efficiency of circRRM2 in BT-549 and MDA-MB-231 was verified by RT qPCR. (C) The levels of IGF2BP1 in BC cells. (D) Expression level of MYC in BC tissues. (E) Correlation analysis of circRRM2 and MYC in BC tissues. (F-G) circRRM2 knockdown abolished the suppression of cell migration treated with miR-27b-3p/miR-31-5p inhibitor. BT-549 cells were transfected with miR-27b-3p (F) or miR-31-5p (G) inhibitor, and the scratch wound healing assay was performed to measure the ability of cell migration. The rescue assay was conducted by co-transfecting the circRRM2 plasmid. *P < 0.05, **P < 0.01, ***P < 0.001. Fig. S7. Prediction and prognostic value of target genes binding with both miR-27b-3p and miR-31-5p. Fig. S8. Prognostic value of target genes binding with both miR-27b-3p and miR-31-5p. *P < 0.05, **P < 0.01, ***P < 0.001. Fig. S9. The transwell assay in BC cell transfected with circRRM2 plasmid and IGF2BP1 siRNA. The transwell assay was performed to detect the rescue effect of overexpression of circRRM2 on IGF2BP1 knockdown in BT-549 (A) or MDA-MB-231 (B) cells. Fig. S10. Calibration plot of the nomogram to predict the probability of the OS in patients wi [file 12935_2023_2895_MOESM1_ESM.zip › 20230321-supplementary materials/20230104-supplementary materials/Supplementary figures/Supplementary figure captions.docx]

**Fig. S1** Analysis of four candidate circRNAs and enrichment analysis of parent genes.

**Fig. S2** The mRNA levels of hsa_circ_0052582, hsa_circ_0058113, hsa_circ_0058148, and hsa_circ_0060551 in patients with BC from the GEO dataset (GSE111504).

**Fig. S3** The mRNA levels of hsa_circ_0052582, hsa_circ_0058113, hsa_circ_0058148, and hsa_circ_0060551 in patients with BC at different T stages from the GEO dataset (GSE111504).

**Fig. S4** The mRNA levels of hsa_circ_0052582, hsa_circ_0058113, hsa_circ_0058148, and hsa_circ_0060551 in patients with BC at different N stages from the GEO dataset (GSE111504).

**Fig. S5** CircRNA-miRNA-mRNA ceRNA network.

**Fig. S6**. Expression level of circRRM2/IGF2BP1/MYC in BC and the migration phenotype of miR-27b-3p/miR-31-5p inhibitor in BC cells. Overexpression (A) or knockout (B) efficiency of circRRM2 in BT-549 and MDA-MB-231 was verified by RT qPCR. (C) The levels of IGF2BP1 in BC cells. (D) Expression level of MYC in BC tissues. (E) Correlation analysis of circRRM2 and MYC in BC tissues. (F-G) circRRM2 knockdown abolished the suppression of cell migration treated with miR-27b-3p/miR-31-5p inhibitor. BT-549 cells were transfected with miR-27b-3p (F) or miR-31-5p (G) inhibitor, and the scratch wound healing assay was performed to measure the ability of cell migration. The rescue assay was conducted by co-transfecting the circRRM2 plasmid. **P* < 0.05, ***P* < 0.01, ****P* < 0.001.

**Fig. S7** Prediction and prognostic value of target genes binding with both miR-27b-3p and miR-31-5p.

**Fig. S8** Prognostic value of target genes binding with both miR-27b-3p and miR-31-5p. **P* < 0.05, ***P* < 0.01, ****P* < 0.001.

**Fig. S9** The transwell assay in BC cell transfected with circRRM2 plasmid and IGF2BP1 siRNA. The transwell assay was performed to detect the rescue effect of overexpression of circRRM2 on IGF2BP1 knockdown in BT-549 (A) or MDA-MB-231 (B) cells.

**Fig. S10** Calibration plot of the nomogram to predict the probability of the OS in patients with BC at 1, 3, and 5 years.

**Fig. S11** Analysis of immune cell infiltration related to IGF2BP1 in patients with BC. (A) Lollipop plot shows the correlation between IGF2BP1 level and immunocytes infiltration. (B) Correlations between IGF2BP1 and immune checkpoint-related genes. (C) Scatter plot of correlation between IGF2BP1 and immunocytes infiltration. (D) KM curve of immunocytes infiltration status and OS in patients with BC. (E) Comparison of immunocytes infiltration related with copy number variation of IGF2BP1. **P* < 0.05, ***P* < 0.01, ****P* < 0.001.

**Fig. S12** Mutational analysis of IGF2BP1. (A) The oncoprint of IGF2BP1 was obtained using cBioPortal. (B) Analysis of PTM sites in IGF2BP1.

**Fig. S13** Network of candidates binding to IGF2BP1 constructed using the BioGRID database.
