# Supplementary material for: A novel feedback regulated loop of circRRM2-IGF2BP1-MYC promotes breast cancer metastasis
Source: Cancer Cell Int. 2023 Mar 25;23:54. doi: 10.1186/s12935-023-02895-w (PMC10039515; doi:10.1186/s12935-023-02895-w)
Supplement: Supplementary file 1 — Additional file 1: Fig. S1. Analysis of four candidate circRNAs and enrichment analysis of parent genes. Fig. S2. The mRNA levels of hsa_circ_0052582, hsa_circ_0058113, hsa_circ_0058148, and hsa_circ_0060551 in patients with BC from the GEO dataset (GSE111504). Fig. S3. The mRNA levels of hsa_circ_0052582, hsa_circ_0058113, hsa_circ_0058148, and hsa_circ_0060551 in patients with BC at different T stages from the GEO dataset (GSE111504). Fig. S4. The mRNA levels of hsa_circ_0052582, hsa_circ_0058113, hsa_circ_0058148, and hsa_circ_0060551 in patients with BC at different N stages from the GEO dataset (GSE111504). Fig. S5. CircRNA-miRNA-mRNA ceRNA network. Fig. S6. Expression level of circRRM2/IGF2BP1/MYC in BC and the migration phenotype of miR-27b-3p/miR-31-5p inhibitor in BC cells. Overexpression (A) or knockout (B) efficiency of circRRM2 in BT-549 and MDA-MB-231 was verified by RT qPCR. (C) The levels of IGF2BP1 in BC cells. (D) Expression level of MYC in BC tissues. (E) Correlation analysis of circRRM2 and MYC in BC tissues. (F-G) circRRM2 knockdown abolished the suppression of cell migration treated with miR-27b-3p/miR-31-5p inhibitor. BT-549 cells were transfected with miR-27b-3p (F) or miR-31-5p (G) inhibitor, and the scratch wound healing assay was performed to measure the ability of cell migration. The rescue assay was conducted by co-transfecting the circRRM2 plasmid. *P < 0.05, **P < 0.01, ***P < 0.001. Fig. S7. Prediction and prognostic value of target genes binding with both miR-27b-3p and miR-31-5p. Fig. S8. Prognostic value of target genes binding with both miR-27b-3p and miR-31-5p. *P < 0.05, **P < 0.01, ***P < 0.001. Fig. S9. The transwell assay in BC cell transfected with circRRM2 plasmid and IGF2BP1 siRNA. The transwell assay was performed to detect the rescue effect of overexpression of circRRM2 on IGF2BP1 knockdown in BT-549 (A) or MDA-MB-231 (B) cells. Fig. S10. Calibration plot of the nomogram to predict the probability of the OS in patients wi [file 12935_2023_2895_MOESM1_ESM.zip › 20230321-supplementary materials/20230104-supplementary materials/Supplementary Tables.docx]

Table S1 Primers used for qRT-PCR.

| Primers | Sequences (5' to 3' ) |
| --- | --- |
| circ58113-F | GCAGTGGTCTTAACAAACATTACTGG |
| circ58113-R | TCCTTGTCATCCTTGACAGTGTAAAC |
| circ58148-F | GACCTATCCAATTTTGGTTCAGACTC |
| circ58148-R | TGGTTGTTGTATAGGAAGGGGAAG |
| circ60551-F | TATGATGTCAGGACCATTCTGCTCT |
| circ60551-R | GGTCTTCATATACCTGTGGGGTTTTT |
| circ52582-F | ACACAAACCATCGGAGGAGAGAGTA |
| circ52582-R | GAGAAATTCCCTTGCTAAAACCCAG |
| MYC-F | CGTCTCCACACATCAGCACAA |
| MYC-R | TCTTGGCAGCAGGATAGTCCTT |
| CTCF-F | TTTGTCTGTTCTAAGTGTGGGAAA |
| CTCF-R | TTAGAGCGCATCTTTCTTTTTCTT |
| JUNB-F | GCACTAAAATGGAACAGCCCT T |
| JUNB-R | GGCTCGGTTTCAGGAGTTTG |
| IGF2BP1-F | AGTGTGCTGGGAGAAGAGGAAG |
| IGF2BP1-R | TTGCGGTTGTCTTGTTGTTACTGTTG |
| GAPDH-F | ACAACTTTGGTATCGTGGAAGG |
| GAPDH-R | GCCATCACGCCACAGTTTC |

Table S2 The sequences of siRNAs.

| siRNAs | Sequences (5' to 3' ) |
| --- | --- |
| Negative control-S | UUCUCCGAACGUGUCACGUTT |
| Negative control-AS | ACGUGACACGUUCGGAGAATT |
| has-miR-27b-3p mimics-S | UUCACAGUGGCUAAGUUCUGC |
| has-miR-27b-3p mimics-AS | AGAACUUAGCCACUGUGAAUU |
| has-miR-31-5p mimics-S | AGGCAAGAUGCUGGCAUAGCU |
| has-miR-31-5p mimics-AS | CUAUGCCAGCAUCUUGCCUUU |
| MircoRNA inhibitor NC | CAGUACUUUUGUGUAGUACAA |
| has-miR-27b-3p inhibitor | GCAGAACUUAGCCACUGUGAA |
| has-miR-31-5p inhibitor | AGCUAUGCCAGCAUCUUGCCU |
| si-circRRM2-S | GGUUUUAGCAAGGGAAUUUTT |
| si-circRRM2-AS | AAAUUCCCUUGCUAAAACCTT |
| si-IGF2BP1-1-S | GGCCAGUUCUUGGUCAAAUTT |
| si-IGF2BP1-1-AS | AUUUGACCAAGAACUGGCCTT |
| si-IGF2BP1-2-S | CCACCAUGAACAAGCUUUATT |
| si-IGF2BP1-2-AS | UAAAGCUUGUUCAUGGUGGTT |
| si-JUNB-S | CGACUACAAACUCCUGAAATT |
| si-JUNB-AS | UUUCAGGAGUUUGUAGUCGTT |
| si-MYC-S | GAGGAUAUCUGGAAGAAAUTT |
| si-MYC-AS | AUUUCUUCCAGAUAUCCUCTT |
| si-CTCF-S | GUAGAAGUCAGCAAAUUAATT |
| si-CTCF-AS | UUAAUUUGCUGACUUCUACTT |

Table S3 Overall characteristics of breast cancer patients.

| Characteristic | Level | Overall (%) |
| --- | --- | --- |
| N |  | 125 |
| Age, median (range) |  | 52.2 (27, 77) |
| Gender, female (%) |  | 126 (100%) |
| T stage | T1 | 27 (21.6%) |
|  | T2 | 86 (68.8%) |
|  | T3 | 5 (4%) |
|  | T4 | 7 (5.6%) |
| N stage | N0 | 76 (60.8%) |
|  | N1 | 37 (29.6%) |
|  | N2 | 4 (3.2%) |
|  | N3 | 8 (6.4%) |
| M stage | M0 | 123 (98.4%) |
|  | M1 | 2 (1.6%) |
| Pathologic stage | Stage I | 20 (16%) |
|  | Stage II | 88 (70.4%) |
|  | Stage III | 15 (12%) |
|  | Stage IV | 2 (1.6%) |
| HER2 status | Negative | 27 (21.6%) |
|  | Positive | 98 (78.4%) |
| ER status | Negative | 32 (25.6%) |
|  | Positive | 93 (74.4%) |
| PR status | Negative | 40 (32%) |
|  | Positive | 85 (68%) |
| P53 | WT | 20 (16%) |
|  | Mutant | 105 (84%) |
| Ki67 | High | 44 (35.2%) |
|  | Low | 81 (64.8%) |

Table S4 GO enrichment entries of biological process (the top 10).

| ID | Description | GeneRatio | BgRatio | *P-*value | *P*adjust | *q-*value | Count |
| --- | --- | --- | --- | --- | --- | --- | --- |
| GO:0000280 | nuclear division | 61/394 | 428/18866 | 2.86E-33 | 1.18E-29 | 9.20E-30 | 61 |
| GO:0140014 | mitotic nuclear division | 50/394 | 286/18866 | 1.27E-31 | 2.16E-28 | 1.68E-28 | 50 |
| GO:0048285 | organelle fission | 62/394 | 476/18866 | 1.57E-31 | 2.16E-28 | 1.68E-28 | 62 |
| GO:0000070 | mitotic sister chromatid segregation | 37/394 | 161/18866 | 4.45E-28 | 4.59E-25 | 3.57E-25 | 37 |
| GO:0000819 | sister chromatid segregation | 38/394 | 196/18866 | 6.43E-26 | 5.30E-23 | 4.13E-23 | 38 |
| GO:0098813 | nuclear chromosome segregation | 41/394 | 272/18866 | 1.76E-23 | 1.21E-20 | 9.43E-21 | 41 |
| GO:0007059 | chromosome segregation | 44/394 | 334/18866 | 1.04E-22 | 6.14E-20 | 4.78E-20 | 44 |
| GO:0051983 | regulation of chromosome segregation | 24/394 | 107/18866 | 2.43E-18 | 1.25E-15 | 9.73E-16 | 24 |
| GO:0007088 | regulation of mitotic nuclear division | 28/394 | 163/18866 | 6.49E-18 | 2.97E-15 | 2.32E-15 | 28 |
| GO:0007052 | mitotic spindle organization | 24/394 | 118/18866 | 2.73E-17 | 1.06E-14 | 8.23E-15 | 24 |

Table S5 GO enrichment entries of cellular components (the top 10).

| ID | Description | GeneRatio | BgRatio | *P-*value | *P*adjust | *q-*value | Count |
| --- | --- | --- | --- | --- | --- | --- | --- |
| GO:0005819 | spindle | 39/408 | 367/19559 | 6.19E-17 | 2.47E-14 | 1.94E-14 | 39 |
| GO:0062023 | collagen-containing extracellular matrix | 40/408 | 427/19559 | 1.88E-15 | 3.76E-13 | 2.94E-13 | 40 |
| GO:0098687 | chromosomal region | 36/408 | 350/19559 | 2.88E-15 | 3.83E-13 | 3.00E-13 | 36 |
| GO:0000793 | condensed chromosome | 27/408 | 222/19559 | 1.72E-13 | 1.71E-11 | 1.34E-11 | 27 |
| GO:0000775 | chromosome, centromeric region | 24/408 | 196/19559 | 3.38E-12 | 2.70E-10 | 2.11E-10 | 24 |
| GO:0000776 | kinetochore | 20/408 | 137/19559 | 8.51E-12 | 5.03E-10 | 3.94E-10 | 20 |
| GO:0000779 | condensed chromosome, centromeric region | 19/408 | 122/19559 | 8.82E-12 | 5.03E-10 | 3.94E-10 | 19 |
| GO:0072686 | mitotic spindle | 19/408 | 133/19559 | 4.19E-11 | 2.09E-09 | 1.64E-09 | 19 |
| GO:0000777 | condensed chromosome kinetochore | 17/408 | 106/19559 | 6.89E-11 | 3.05E-09 | 2.39E-09 | 17 |
| GO:0030496 | midbody | 20/408 | 182/19559 | 1.54E-09 | 6.13E-08 | 4.80E-08 | 20 |

Table S6 GO enrichment entries of molecular function (the top 10).

| ID | Description | GeneRatio | BgRatio | *P-*value | *P*adjust | *q-*value | Count |
| --- | --- | --- | --- | --- | --- | --- | --- |
| GO:0015631 | tubulin binding | 29/393 | 365/18352 | 1.45E-09 | 2.35E-07 | 1.95E-07 | 29 |
| GO:0016887 | ATPase activity | 28/393 | 423/18352 | 1.41E-07 | 1.53E-05 | 1.26E-05 | 28 |
| GO:0008017 | microtubule binding | 27/393 | 265/18352 | 2.09E-11 | 6.77E-09 | 5.61E-09 | 27 |
| GO:0005201 | extracellular matrix structural constituent | 24/393 | 169/18352 | 2.16E-13 | 1.40E-10 | 1.16E-10 | 24 |
| GO:0004674 | protein serine/threonine kinase activity | 23/393 | 435/18352 | 6.94E-05 | 0.0018747 | 0.0015531 | 23 |
| GO:0003779 | actin binding | 21/393 | 437/18352 | 0.0005137 | 0.00951 | 0.0078786 | 21 |
| GO:0005539 | glycosaminoglycan binding | 19/393 | 232/18352 | 6.55E-07 | 4.72E-05 | 3.91E-05 | 19 |
| GO:1901681 | sulfur compound binding | 17/393 | 262/18352 | 5.34E-05 | 0.0016463 | 0.0013639 | 17 |
| GO:0140097 | catalytic activity, acting on DNA | 16/393 | 209/18352 | 1.18E-05 | 0.0005888 | 0.0004878 | 16 |
| GO:0019199 | transmembrane receptor protein kinase activity | 15/393 | 80/18352 | 1.31E-10 | 2.83E-08 | 2.34E-08 | 15 |

Table S7 Top 10 enriched KEGG pathways.

| ID | Description | GeneRatio | BgRatio | *P-*value | *P*adjust | *q-*value | Count |
| --- | --- | --- | --- | --- | --- | --- | --- |
| hsa04110 | Cell cycle | 21/194 | 126/8146 | 1.35E-12 | 3.57E-10 | 3.23E-10 | 21 |
| hsa04114 | Oocyte meiosis | 15/194 | 131/8146 | 4.43E-07 | 5.84E-05 | 5.29E-05 | 15 |
| hsa05166 | Human T-cell leukemia virus 1 infection | 15/194 | 222/8146 | 2.56E-04 | 0.011776 | 0.0106585 | 15 |
| hsa05165 | Human papillomavirus infection | 14/194 | 331/8146 | 0.026397 | 0.2670621 | 0.2417188 | 14 |
| hsa04151 | PI3K-Akt signaling pathway | 14/194 | 354/8146 | 0.0429016 | 0.3331185 | 0.3015068 | 14 |
| hsa04218 | Cellular senescence | 13/194 | 156/8146 | 8.36E-05 | 0.0055158 | 0.0049924 | 13 |
| hsa04914 | Progesterone-mediated oocyte maturation | 12/194 | 102/8146 | 4.91E-06 | 4.32E-04 | 3.91E-04 | 12 |
| hsa04010 | MAPK signaling pathway | 12/194 | 294/8146 | 0.0480222 | 0.342645 | 0.3101292 | 12 |
| hsa05206 | MicroRNAs in cancer | 12/194 | 310/8146 | 0.0663305 | 0.3594691 | 0.3253568 | 12 |
| hsa04360 | Axon guidance | 11/194 | 182/8146 | 0.0040743 | 0.0977836 | 0.0885043 | 11 |
