# Supplementary figures and images for: A novel feedback regulated loop of circRRM2-IGF2BP1-MYC promotes breast cancer metastasis
Source: Cancer Cell Int. 2023 Mar 25;23:54. doi: 10.1186/s12935-023-02895-w (PMC10039515; doi:10.1186/s12935-023-02895-w)

**A**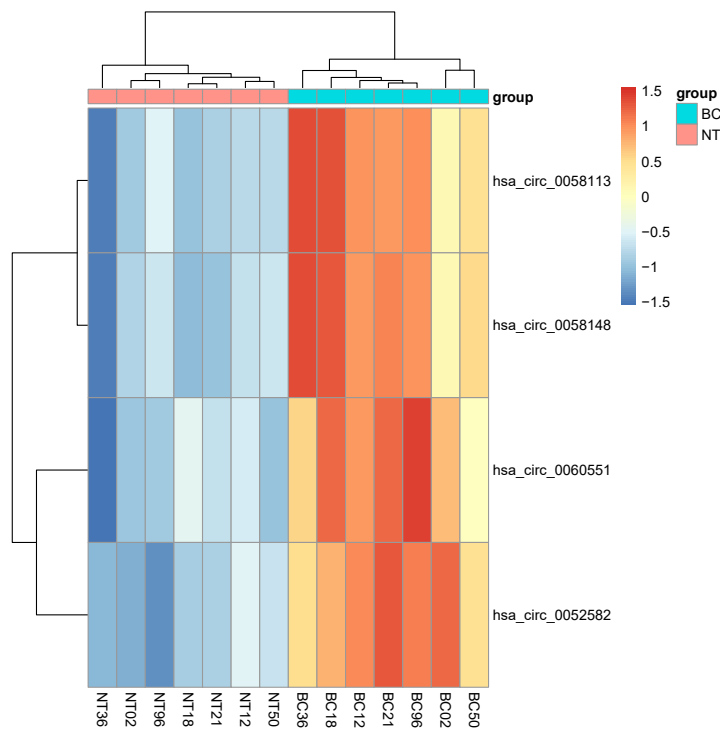**B**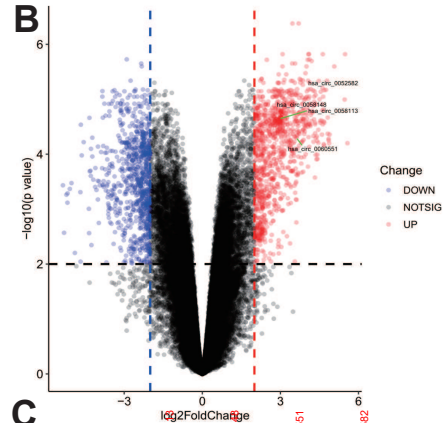**C**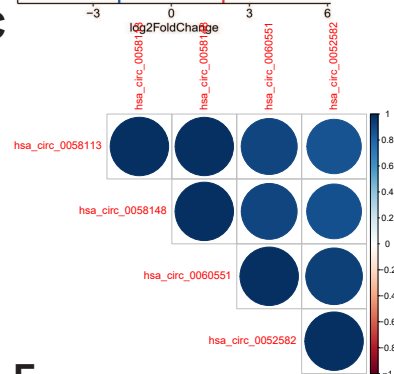**D**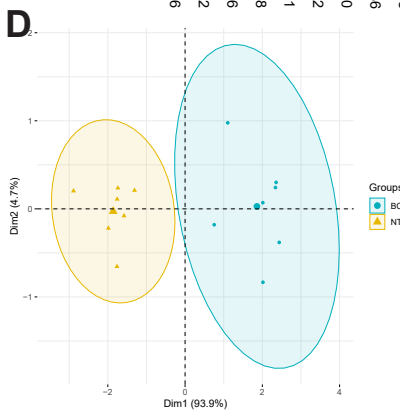**E**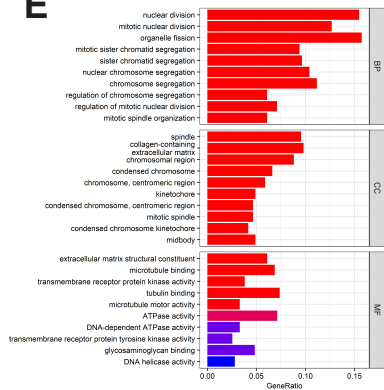**F**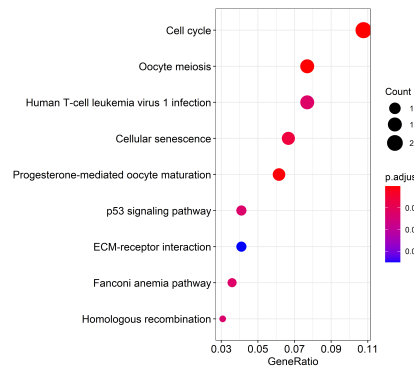

Supplement: Supplementary file 1 — Additional file 1: Fig. S1. Analysis of four candidate circRNAs and enrichment analysis of parent genes. Fig. S2. The mRNA levels of hsa_circ_0052582, hsa_circ_0058113, hsa_circ_0058148, and hsa_circ_0060551 in patients with BC from the GEO dataset (GSE111504). Fig. S3. The mRNA levels of hsa_circ_0052582, hsa_circ_0058113, hsa_circ_0058148, and hsa_circ_0060551 in patients with BC at different T stages from the GEO dataset (GSE111504). Fig. S4. The mRNA levels of hsa_circ_0052582, hsa_circ_0058113, hsa_circ_0058148, and hsa_circ_0060551 in patients with BC at different N stages from the GEO dataset (GSE111504). Fig. S5. CircRNA-miRNA-mRNA ceRNA network. Fig. S6. Expression level of circRRM2/IGF2BP1/MYC in BC and the migration phenotype of miR-27b-3p/miR-31-5p inhibitor in BC cells. Overexpression (A) or knockout (B) efficiency of circRRM2 in BT-549 and MDA-MB-231 was verified by RT qPCR. (C) The levels of IGF2BP1 in BC cells. (D) Expression level of MYC in BC tissues. (E) Correlation analysis of circRRM2 and MYC in BC tissues. (F-G) circRRM2 knockdown abolished the suppression of cell migration treated with miR-27b-3p/miR-31-5p inhibitor. BT-549 cells were transfected with miR-27b-3p (F) or miR-31-5p (G) inhibitor, and the scratch wound healing assay was performed to measure the ability of cell migration. The rescue assay was conducted by co-transfecting the circRRM2 plasmid. *P < 0.05, **P < 0.01, ***P < 0.001. Fig. S7. Prediction and prognostic value of target genes binding with both miR-27b-3p and miR-31-5p. Fig. S8. Prognostic value of target genes binding with both miR-27b-3p and miR-31-5p. *P < 0.05, **P < 0.01, ***P < 0.001. Fig. S9. The transwell assay in BC cell transfected with circRRM2 plasmid and IGF2BP1 siRNA. The transwell assay was performed to detect the rescue effect of overexpression of circRRM2 on IGF2BP1 knockdown in BT-549 (A) or MDA-MB-231 (B) cells. Fig. S10. Calibration plot of the nomogram to predict the probability of the OS in patients wi [file 12935_2023_2895_MOESM1_ESM.zip › 20230321-supplementary materials/20230104-supplementary materials/Supplementary figures/Figure S1.pdf]

Actual 1-Year Survival

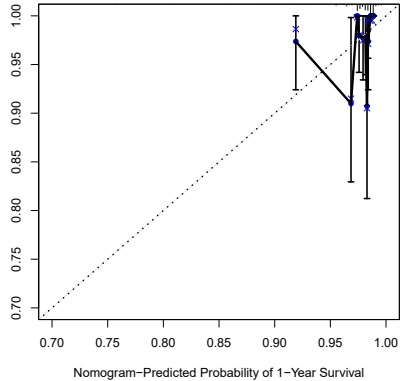

Actual 3-Year Survival

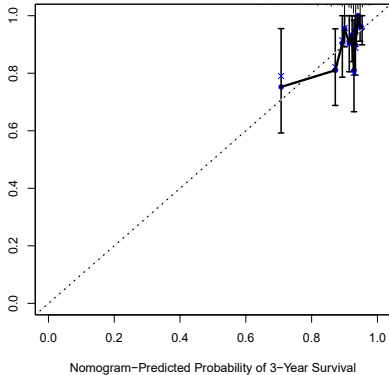

Actual 5-Year Survival

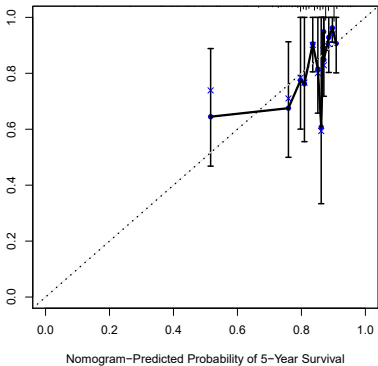

Supplement: Supplementary file 1 — Additional file 1: Fig. S1. Analysis of four candidate circRNAs and enrichment analysis of parent genes. Fig. S2. The mRNA levels of hsa_circ_0052582, hsa_circ_0058113, hsa_circ_0058148, and hsa_circ_0060551 in patients with BC from the GEO dataset (GSE111504). Fig. S3. The mRNA levels of hsa_circ_0052582, hsa_circ_0058113, hsa_circ_0058148, and hsa_circ_0060551 in patients with BC at different T stages from the GEO dataset (GSE111504). Fig. S4. The mRNA levels of hsa_circ_0052582, hsa_circ_0058113, hsa_circ_0058148, and hsa_circ_0060551 in patients with BC at different N stages from the GEO dataset (GSE111504). Fig. S5. CircRNA-miRNA-mRNA ceRNA network. Fig. S6. Expression level of circRRM2/IGF2BP1/MYC in BC and the migration phenotype of miR-27b-3p/miR-31-5p inhibitor in BC cells. Overexpression (A) or knockout (B) efficiency of circRRM2 in BT-549 and MDA-MB-231 was verified by RT qPCR. (C) The levels of IGF2BP1 in BC cells. (D) Expression level of MYC in BC tissues. (E) Correlation analysis of circRRM2 and MYC in BC tissues. (F-G) circRRM2 knockdown abolished the suppression of cell migration treated with miR-27b-3p/miR-31-5p inhibitor. BT-549 cells were transfected with miR-27b-3p (F) or miR-31-5p (G) inhibitor, and the scratch wound healing assay was performed to measure the ability of cell migration. The rescue assay was conducted by co-transfecting the circRRM2 plasmid. *P < 0.05, **P < 0.01, ***P < 0.001. Fig. S7. Prediction and prognostic value of target genes binding with both miR-27b-3p and miR-31-5p. Fig. S8. Prognostic value of target genes binding with both miR-27b-3p and miR-31-5p. *P < 0.05, **P < 0.01, ***P < 0.001. Fig. S9. The transwell assay in BC cell transfected with circRRM2 plasmid and IGF2BP1 siRNA. The transwell assay was performed to detect the rescue effect of overexpression of circRRM2 on IGF2BP1 knockdown in BT-549 (A) or MDA-MB-231 (B) cells. Fig. S10. Calibration plot of the nomogram to predict the probability of the OS in patients wi [file 12935_2023_2895_MOESM1_ESM.zip › 20230321-supplementary materials/20230104-supplementary materials/Supplementary figures/Figure S10.pdf]

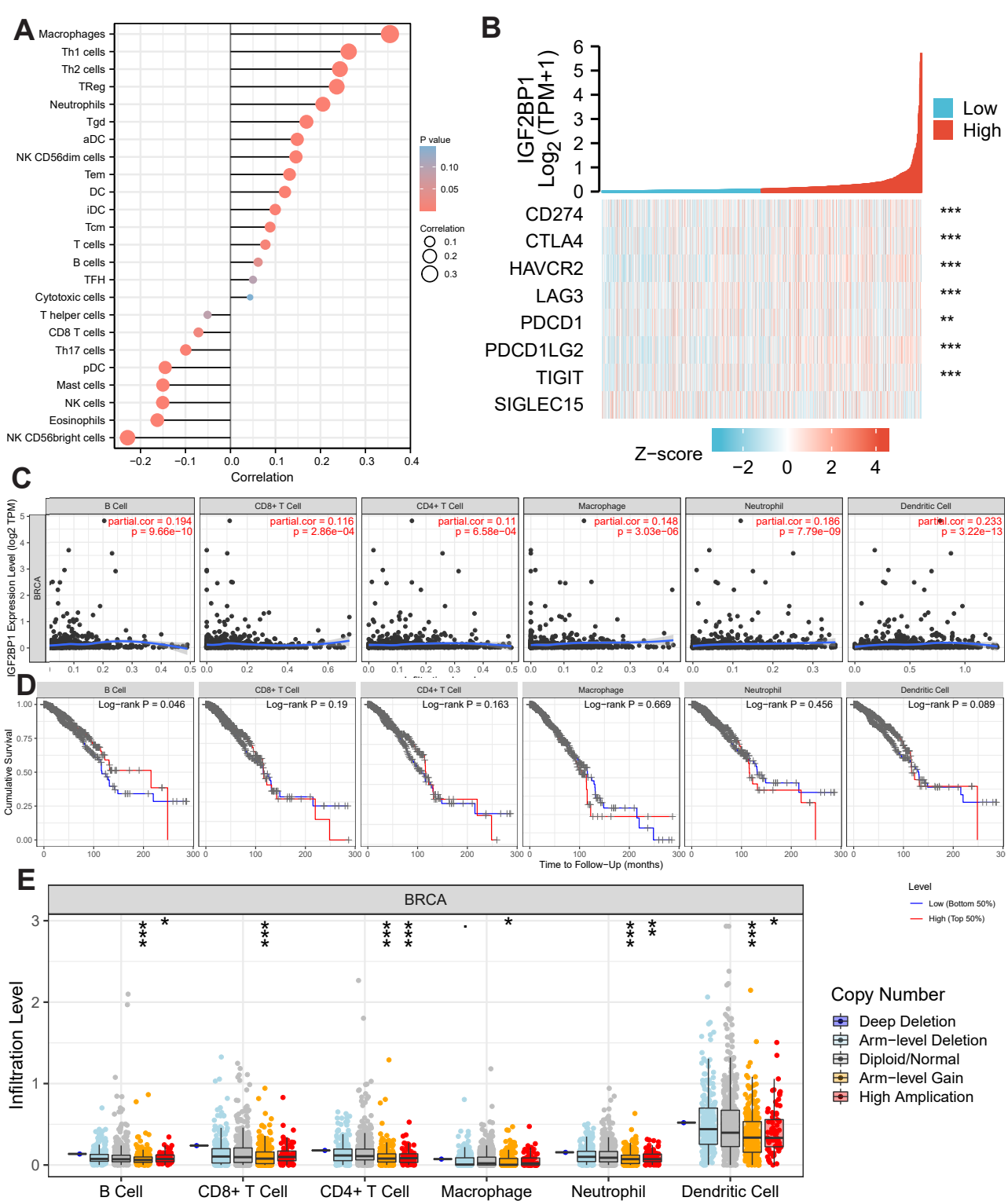

Supplement: Supplementary file 1 — Additional file 1: Fig. S1. Analysis of four candidate circRNAs and enrichment analysis of parent genes. Fig. S2. The mRNA levels of hsa_circ_0052582, hsa_circ_0058113, hsa_circ_0058148, and hsa_circ_0060551 in patients with BC from the GEO dataset (GSE111504). Fig. S3. The mRNA levels of hsa_circ_0052582, hsa_circ_0058113, hsa_circ_0058148, and hsa_circ_0060551 in patients with BC at different T stages from the GEO dataset (GSE111504). Fig. S4. The mRNA levels of hsa_circ_0052582, hsa_circ_0058113, hsa_circ_0058148, and hsa_circ_0060551 in patients with BC at different N stages from the GEO dataset (GSE111504). Fig. S5. CircRNA-miRNA-mRNA ceRNA network. Fig. S6. Expression level of circRRM2/IGF2BP1/MYC in BC and the migration phenotype of miR-27b-3p/miR-31-5p inhibitor in BC cells. Overexpression (A) or knockout (B) efficiency of circRRM2 in BT-549 and MDA-MB-231 was verified by RT qPCR. (C) The levels of IGF2BP1 in BC cells. (D) Expression level of MYC in BC tissues. (E) Correlation analysis of circRRM2 and MYC in BC tissues. (F-G) circRRM2 knockdown abolished the suppression of cell migration treated with miR-27b-3p/miR-31-5p inhibitor. BT-549 cells were transfected with miR-27b-3p (F) or miR-31-5p (G) inhibitor, and the scratch wound healing assay was performed to measure the ability of cell migration. The rescue assay was conducted by co-transfecting the circRRM2 plasmid. *P < 0.05, **P < 0.01, ***P < 0.001. Fig. S7. Prediction and prognostic value of target genes binding with both miR-27b-3p and miR-31-5p. Fig. S8. Prognostic value of target genes binding with both miR-27b-3p and miR-31-5p. *P < 0.05, **P < 0.01, ***P < 0.001. Fig. S9. The transwell assay in BC cell transfected with circRRM2 plasmid and IGF2BP1 siRNA. The transwell assay was performed to detect the rescue effect of overexpression of circRRM2 on IGF2BP1 knockdown in BT-549 (A) or MDA-MB-231 (B) cells. Fig. S10. Calibration plot of the nomogram to predict the probability of the OS in patients wi [file 12935_2023_2895_MOESM1_ESM.zip › 20230321-supplementary materials/20230104-supplementary materials/Supplementary figures/Figure S11.pdf]

A

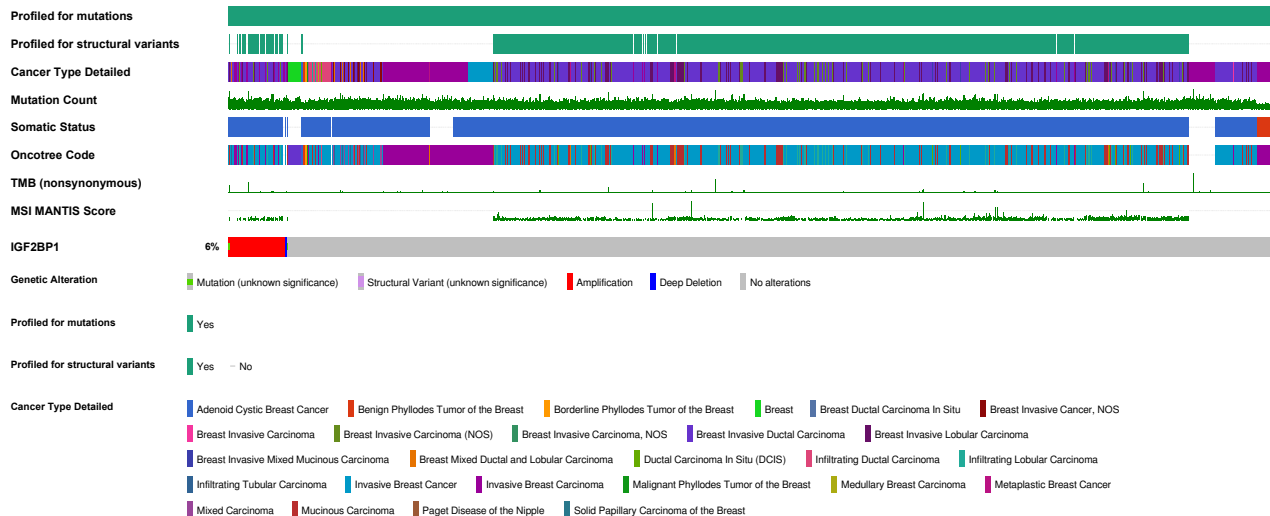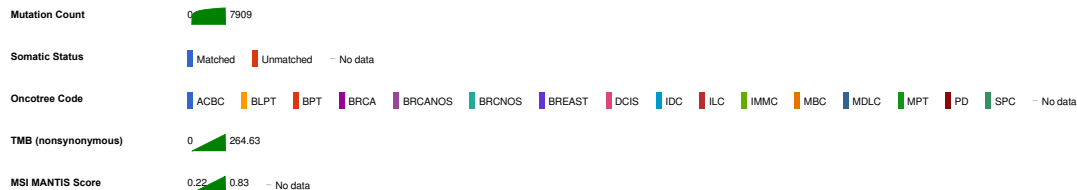

B

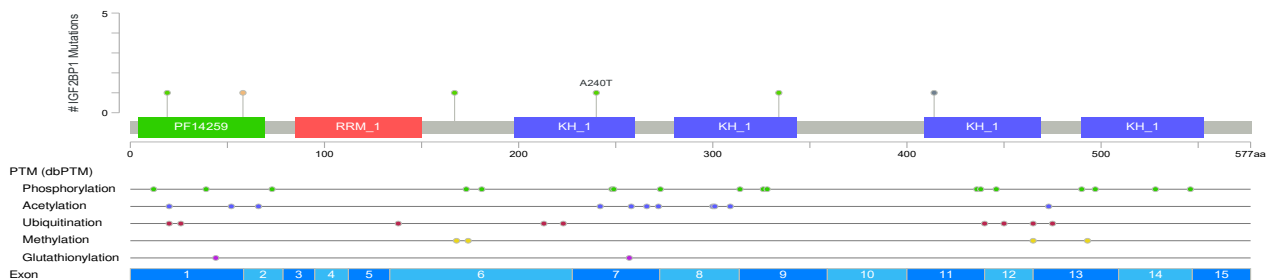

Supplement: Supplementary file 1 — Additional file 1: Fig. S1. Analysis of four candidate circRNAs and enrichment analysis of parent genes. Fig. S2. The mRNA levels of hsa_circ_0052582, hsa_circ_0058113, hsa_circ_0058148, and hsa_circ_0060551 in patients with BC from the GEO dataset (GSE111504). Fig. S3. The mRNA levels of hsa_circ_0052582, hsa_circ_0058113, hsa_circ_0058148, and hsa_circ_0060551 in patients with BC at different T stages from the GEO dataset (GSE111504). Fig. S4. The mRNA levels of hsa_circ_0052582, hsa_circ_0058113, hsa_circ_0058148, and hsa_circ_0060551 in patients with BC at different N stages from the GEO dataset (GSE111504). Fig. S5. CircRNA-miRNA-mRNA ceRNA network. Fig. S6. Expression level of circRRM2/IGF2BP1/MYC in BC and the migration phenotype of miR-27b-3p/miR-31-5p inhibitor in BC cells. Overexpression (A) or knockout (B) efficiency of circRRM2 in BT-549 and MDA-MB-231 was verified by RT qPCR. (C) The levels of IGF2BP1 in BC cells. (D) Expression level of MYC in BC tissues. (E) Correlation analysis of circRRM2 and MYC in BC tissues. (F-G) circRRM2 knockdown abolished the suppression of cell migration treated with miR-27b-3p/miR-31-5p inhibitor. BT-549 cells were transfected with miR-27b-3p (F) or miR-31-5p (G) inhibitor, and the scratch wound healing assay was performed to measure the ability of cell migration. The rescue assay was conducted by co-transfecting the circRRM2 plasmid. *P < 0.05, **P < 0.01, ***P < 0.001. Fig. S7. Prediction and prognostic value of target genes binding with both miR-27b-3p and miR-31-5p. Fig. S8. Prognostic value of target genes binding with both miR-27b-3p and miR-31-5p. *P < 0.05, **P < 0.01, ***P < 0.001. Fig. S9. The transwell assay in BC cell transfected with circRRM2 plasmid and IGF2BP1 siRNA. The transwell assay was performed to detect the rescue effect of overexpression of circRRM2 on IGF2BP1 knockdown in BT-549 (A) or MDA-MB-231 (B) cells. Fig. S10. Calibration plot of the nomogram to predict the probability of the OS in patients wi [file 12935_2023_2895_MOESM1_ESM.zip › 20230321-supplementary materials/20230104-supplementary materials/Supplementary figures/Figure S12.pdf]

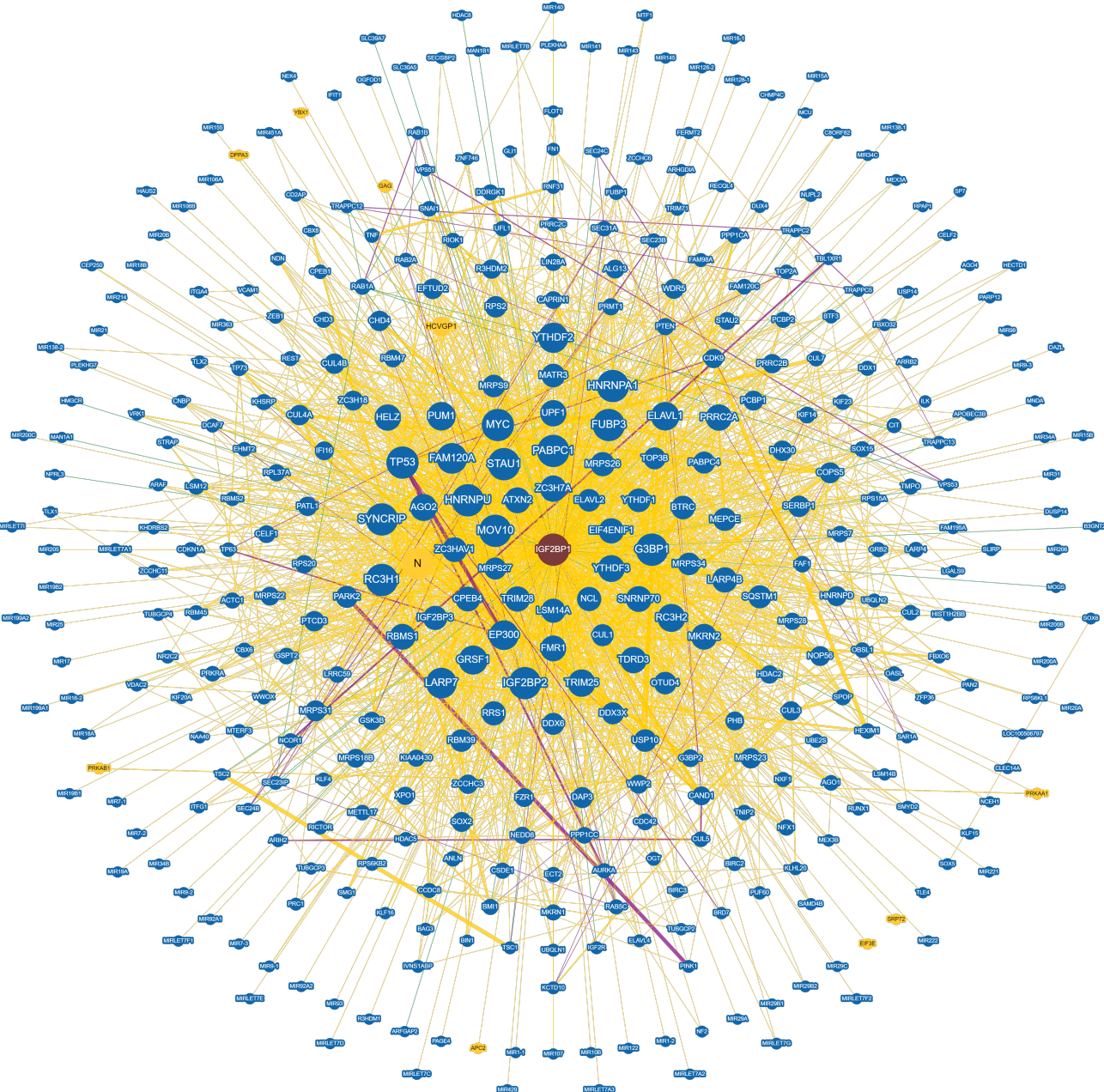

Supplement: Supplementary file 1 — Additional file 1: Fig. S1. Analysis of four candidate circRNAs and enrichment analysis of parent genes. Fig. S2. The mRNA levels of hsa_circ_0052582, hsa_circ_0058113, hsa_circ_0058148, and hsa_circ_0060551 in patients with BC from the GEO dataset (GSE111504). Fig. S3. The mRNA levels of hsa_circ_0052582, hsa_circ_0058113, hsa_circ_0058148, and hsa_circ_0060551 in patients with BC at different T stages from the GEO dataset (GSE111504). Fig. S4. The mRNA levels of hsa_circ_0052582, hsa_circ_0058113, hsa_circ_0058148, and hsa_circ_0060551 in patients with BC at different N stages from the GEO dataset (GSE111504). Fig. S5. CircRNA-miRNA-mRNA ceRNA network. Fig. S6. Expression level of circRRM2/IGF2BP1/MYC in BC and the migration phenotype of miR-27b-3p/miR-31-5p inhibitor in BC cells. Overexpression (A) or knockout (B) efficiency of circRRM2 in BT-549 and MDA-MB-231 was verified by RT qPCR. (C) The levels of IGF2BP1 in BC cells. (D) Expression level of MYC in BC tissues. (E) Correlation analysis of circRRM2 and MYC in BC tissues. (F-G) circRRM2 knockdown abolished the suppression of cell migration treated with miR-27b-3p/miR-31-5p inhibitor. BT-549 cells were transfected with miR-27b-3p (F) or miR-31-5p (G) inhibitor, and the scratch wound healing assay was performed to measure the ability of cell migration. The rescue assay was conducted by co-transfecting the circRRM2 plasmid. *P < 0.05, **P < 0.01, ***P < 0.001. Fig. S7. Prediction and prognostic value of target genes binding with both miR-27b-3p and miR-31-5p. Fig. S8. Prognostic value of target genes binding with both miR-27b-3p and miR-31-5p. *P < 0.05, **P < 0.01, ***P < 0.001. Fig. S9. The transwell assay in BC cell transfected with circRRM2 plasmid and IGF2BP1 siRNA. The transwell assay was performed to detect the rescue effect of overexpression of circRRM2 on IGF2BP1 knockdown in BT-549 (A) or MDA-MB-231 (B) cells. Fig. S10. Calibration plot of the nomogram to predict the probability of the OS in patients wi [file 12935_2023_2895_MOESM1_ESM.zip › 20230321-supplementary materials/20230104-supplementary materials/Supplementary figures/Figure S13.pdf]

Group NT BC

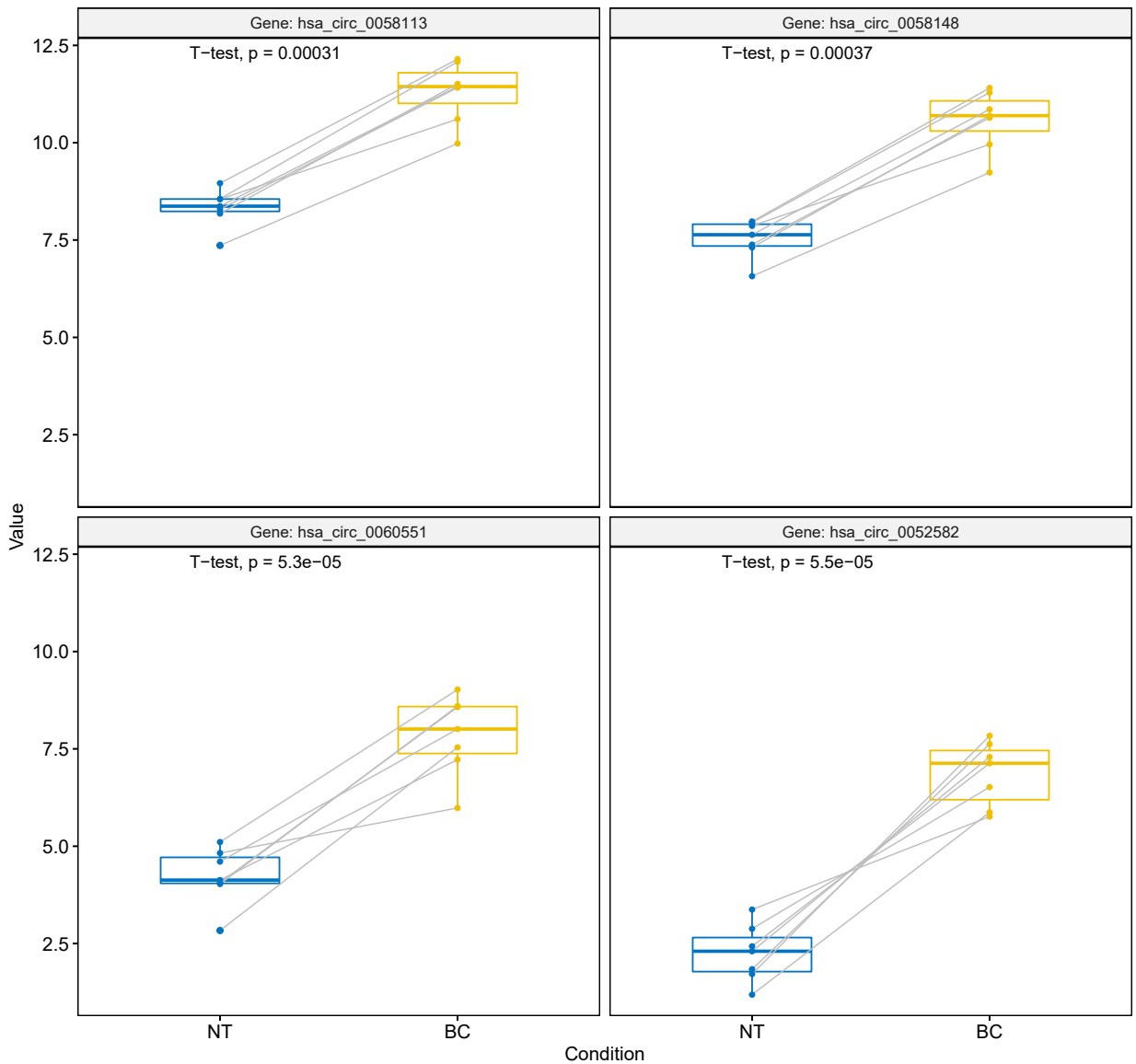

Supplement: Supplementary file 1 — Additional file 1: Fig. S1. Analysis of four candidate circRNAs and enrichment analysis of parent genes. Fig. S2. The mRNA levels of hsa_circ_0052582, hsa_circ_0058113, hsa_circ_0058148, and hsa_circ_0060551 in patients with BC from the GEO dataset (GSE111504). Fig. S3. The mRNA levels of hsa_circ_0052582, hsa_circ_0058113, hsa_circ_0058148, and hsa_circ_0060551 in patients with BC at different T stages from the GEO dataset (GSE111504). Fig. S4. The mRNA levels of hsa_circ_0052582, hsa_circ_0058113, hsa_circ_0058148, and hsa_circ_0060551 in patients with BC at different N stages from the GEO dataset (GSE111504). Fig. S5. CircRNA-miRNA-mRNA ceRNA network. Fig. S6. Expression level of circRRM2/IGF2BP1/MYC in BC and the migration phenotype of miR-27b-3p/miR-31-5p inhibitor in BC cells. Overexpression (A) or knockout (B) efficiency of circRRM2 in BT-549 and MDA-MB-231 was verified by RT qPCR. (C) The levels of IGF2BP1 in BC cells. (D) Expression level of MYC in BC tissues. (E) Correlation analysis of circRRM2 and MYC in BC tissues. (F-G) circRRM2 knockdown abolished the suppression of cell migration treated with miR-27b-3p/miR-31-5p inhibitor. BT-549 cells were transfected with miR-27b-3p (F) or miR-31-5p (G) inhibitor, and the scratch wound healing assay was performed to measure the ability of cell migration. The rescue assay was conducted by co-transfecting the circRRM2 plasmid. *P < 0.05, **P < 0.01, ***P < 0.001. Fig. S7. Prediction and prognostic value of target genes binding with both miR-27b-3p and miR-31-5p. Fig. S8. Prognostic value of target genes binding with both miR-27b-3p and miR-31-5p. *P < 0.05, **P < 0.01, ***P < 0.001. Fig. S9. The transwell assay in BC cell transfected with circRRM2 plasmid and IGF2BP1 siRNA. The transwell assay was performed to detect the rescue effect of overexpression of circRRM2 on IGF2BP1 knockdown in BT-549 (A) or MDA-MB-231 (B) cells. Fig. S10. Calibration plot of the nomogram to predict the probability of the OS in patients wi [file 12935_2023_2895_MOESM1_ESM.zip › 20230321-supplementary materials/20230104-supplementary materials/Supplementary figures/Figure S2.pdf]

NT T1 T2

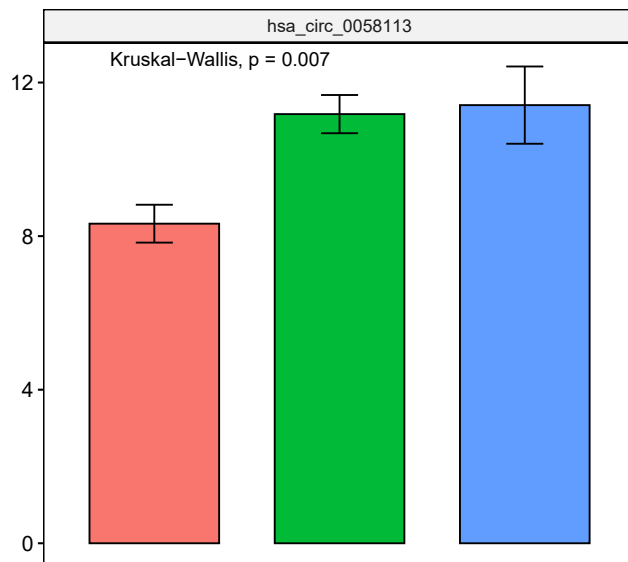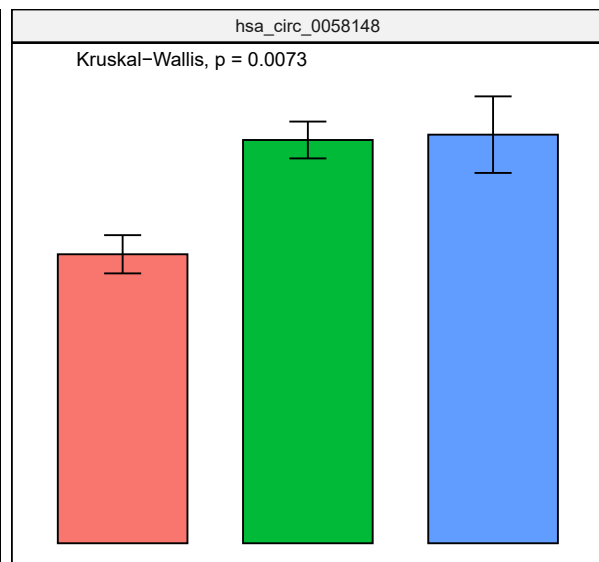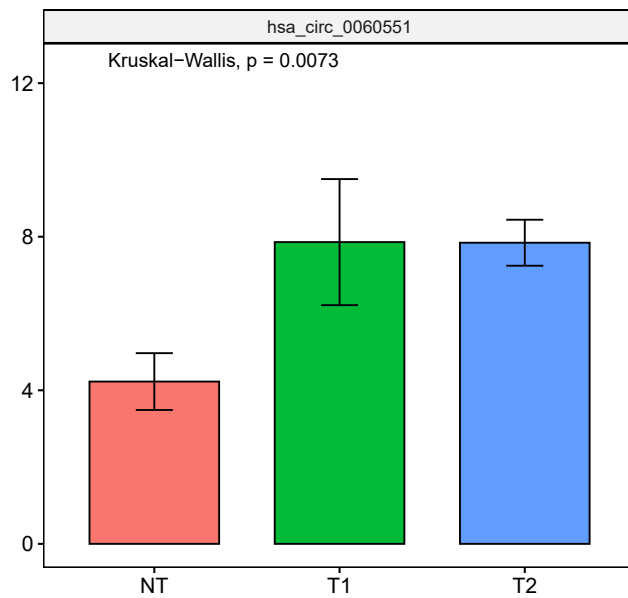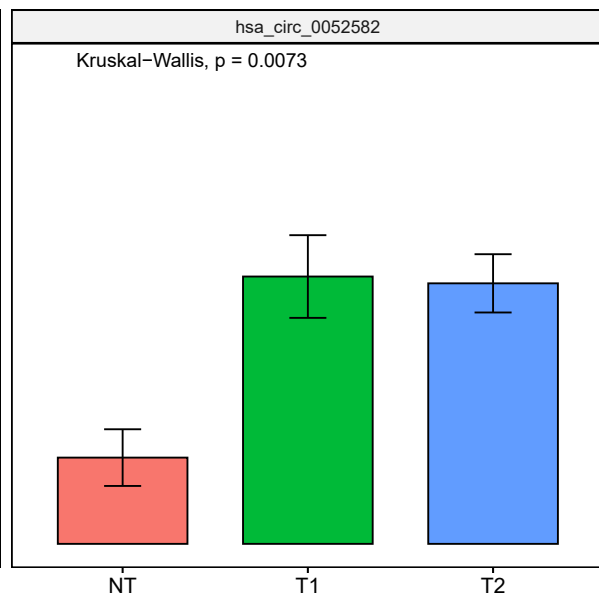

T

Supplement: Supplementary file 1 — Additional file 1: Fig. S1. Analysis of four candidate circRNAs and enrichment analysis of parent genes. Fig. S2. The mRNA levels of hsa_circ_0052582, hsa_circ_0058113, hsa_circ_0058148, and hsa_circ_0060551 in patients with BC from the GEO dataset (GSE111504). Fig. S3. The mRNA levels of hsa_circ_0052582, hsa_circ_0058113, hsa_circ_0058148, and hsa_circ_0060551 in patients with BC at different T stages from the GEO dataset (GSE111504). Fig. S4. The mRNA levels of hsa_circ_0052582, hsa_circ_0058113, hsa_circ_0058148, and hsa_circ_0060551 in patients with BC at different N stages from the GEO dataset (GSE111504). Fig. S5. CircRNA-miRNA-mRNA ceRNA network. Fig. S6. Expression level of circRRM2/IGF2BP1/MYC in BC and the migration phenotype of miR-27b-3p/miR-31-5p inhibitor in BC cells. Overexpression (A) or knockout (B) efficiency of circRRM2 in BT-549 and MDA-MB-231 was verified by RT qPCR. (C) The levels of IGF2BP1 in BC cells. (D) Expression level of MYC in BC tissues. (E) Correlation analysis of circRRM2 and MYC in BC tissues. (F-G) circRRM2 knockdown abolished the suppression of cell migration treated with miR-27b-3p/miR-31-5p inhibitor. BT-549 cells were transfected with miR-27b-3p (F) or miR-31-5p (G) inhibitor, and the scratch wound healing assay was performed to measure the ability of cell migration. The rescue assay was conducted by co-transfecting the circRRM2 plasmid. *P < 0.05, **P < 0.01, ***P < 0.001. Fig. S7. Prediction and prognostic value of target genes binding with both miR-27b-3p and miR-31-5p. Fig. S8. Prognostic value of target genes binding with both miR-27b-3p and miR-31-5p. *P < 0.05, **P < 0.01, ***P < 0.001. Fig. S9. The transwell assay in BC cell transfected with circRRM2 plasmid and IGF2BP1 siRNA. The transwell assay was performed to detect the rescue effect of overexpression of circRRM2 on IGF2BP1 knockdown in BT-549 (A) or MDA-MB-231 (B) cells. Fig. S10. Calibration plot of the nomogram to predict the probability of the OS in patients wi [file 12935_2023_2895_MOESM1_ESM.zip › 20230321-supplementary materials/20230104-supplementary materials/Supplementary figures/Figure S3.pdf]

NT N0 N1

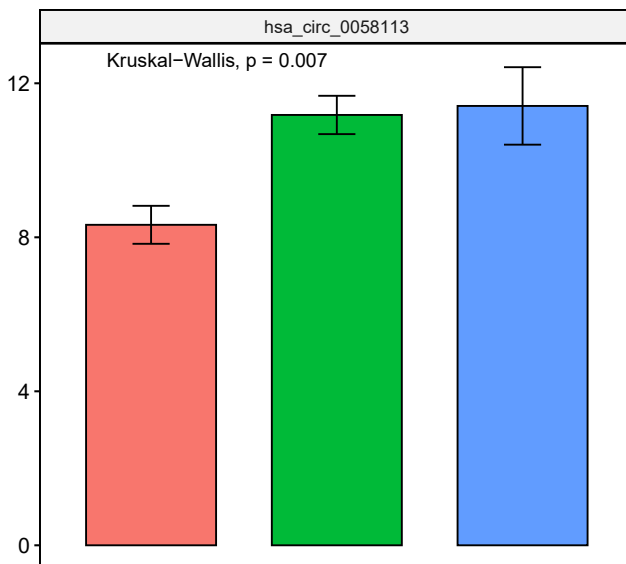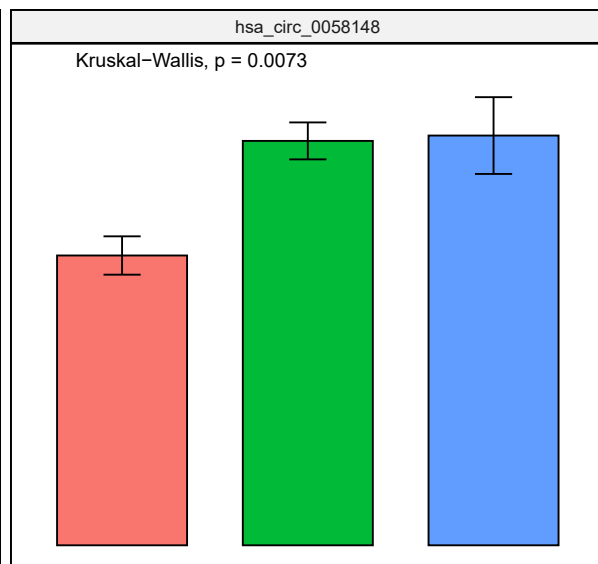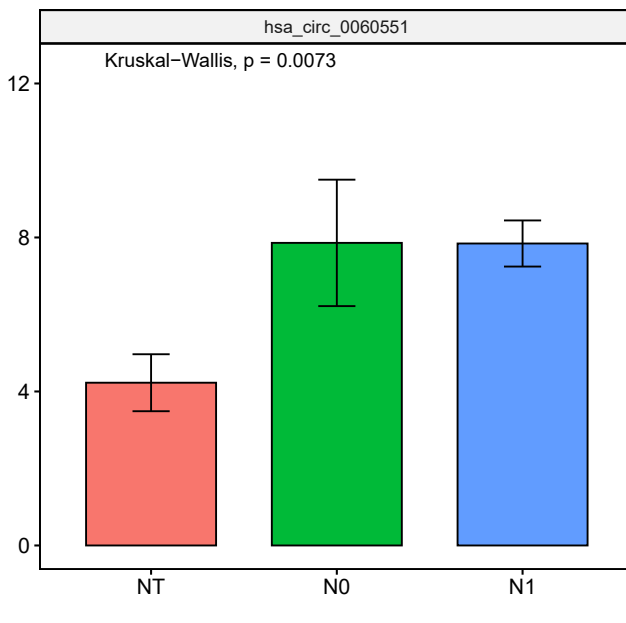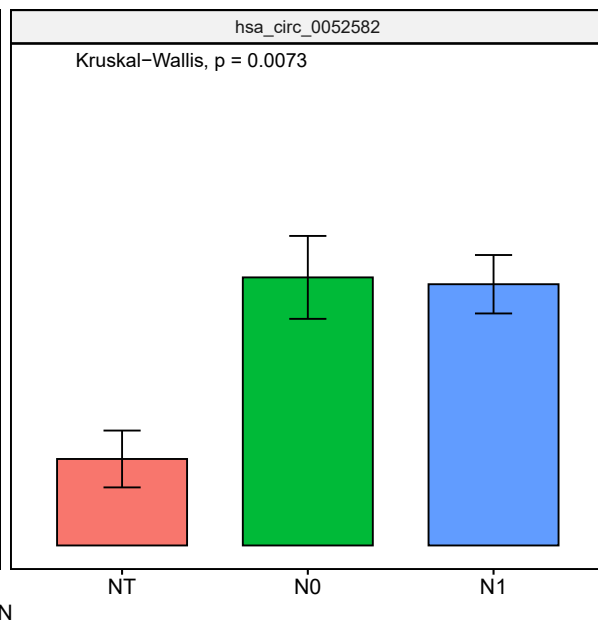

Supplement: Supplementary file 1 — Additional file 1: Fig. S1. Analysis of four candidate circRNAs and enrichment analysis of parent genes. Fig. S2. The mRNA levels of hsa_circ_0052582, hsa_circ_0058113, hsa_circ_0058148, and hsa_circ_0060551 in patients with BC from the GEO dataset (GSE111504). Fig. S3. The mRNA levels of hsa_circ_0052582, hsa_circ_0058113, hsa_circ_0058148, and hsa_circ_0060551 in patients with BC at different T stages from the GEO dataset (GSE111504). Fig. S4. The mRNA levels of hsa_circ_0052582, hsa_circ_0058113, hsa_circ_0058148, and hsa_circ_0060551 in patients with BC at different N stages from the GEO dataset (GSE111504). Fig. S5. CircRNA-miRNA-mRNA ceRNA network. Fig. S6. Expression level of circRRM2/IGF2BP1/MYC in BC and the migration phenotype of miR-27b-3p/miR-31-5p inhibitor in BC cells. Overexpression (A) or knockout (B) efficiency of circRRM2 in BT-549 and MDA-MB-231 was verified by RT qPCR. (C) The levels of IGF2BP1 in BC cells. (D) Expression level of MYC in BC tissues. (E) Correlation analysis of circRRM2 and MYC in BC tissues. (F-G) circRRM2 knockdown abolished the suppression of cell migration treated with miR-27b-3p/miR-31-5p inhibitor. BT-549 cells were transfected with miR-27b-3p (F) or miR-31-5p (G) inhibitor, and the scratch wound healing assay was performed to measure the ability of cell migration. The rescue assay was conducted by co-transfecting the circRRM2 plasmid. *P < 0.05, **P < 0.01, ***P < 0.001. Fig. S7. Prediction and prognostic value of target genes binding with both miR-27b-3p and miR-31-5p. Fig. S8. Prognostic value of target genes binding with both miR-27b-3p and miR-31-5p. *P < 0.05, **P < 0.01, ***P < 0.001. Fig. S9. The transwell assay in BC cell transfected with circRRM2 plasmid and IGF2BP1 siRNA. The transwell assay was performed to detect the rescue effect of overexpression of circRRM2 on IGF2BP1 knockdown in BT-549 (A) or MDA-MB-231 (B) cells. Fig. S10. Calibration plot of the nomogram to predict the probability of the OS in patients wi [file 12935_2023_2895_MOESM1_ESM.zip › 20230321-supplementary materials/20230104-supplementary materials/Supplementary figures/Figure S4.pdf]

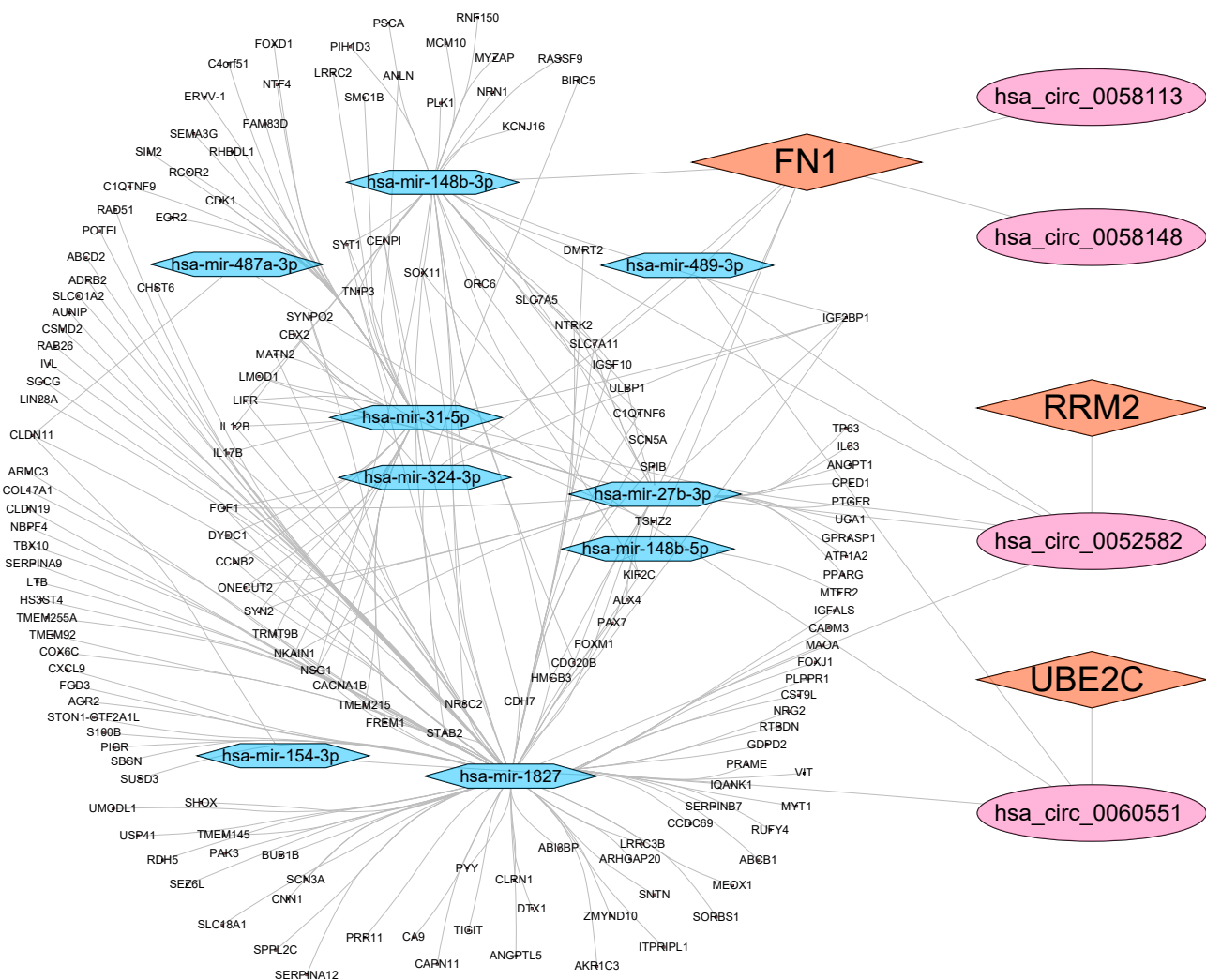

Supplement: Supplementary file 1 — Additional file 1: Fig. S1. Analysis of four candidate circRNAs and enrichment analysis of parent genes. Fig. S2. The mRNA levels of hsa_circ_0052582, hsa_circ_0058113, hsa_circ_0058148, and hsa_circ_0060551 in patients with BC from the GEO dataset (GSE111504). Fig. S3. The mRNA levels of hsa_circ_0052582, hsa_circ_0058113, hsa_circ_0058148, and hsa_circ_0060551 in patients with BC at different T stages from the GEO dataset (GSE111504). Fig. S4. The mRNA levels of hsa_circ_0052582, hsa_circ_0058113, hsa_circ_0058148, and hsa_circ_0060551 in patients with BC at different N stages from the GEO dataset (GSE111504). Fig. S5. CircRNA-miRNA-mRNA ceRNA network. Fig. S6. Expression level of circRRM2/IGF2BP1/MYC in BC and the migration phenotype of miR-27b-3p/miR-31-5p inhibitor in BC cells. Overexpression (A) or knockout (B) efficiency of circRRM2 in BT-549 and MDA-MB-231 was verified by RT qPCR. (C) The levels of IGF2BP1 in BC cells. (D) Expression level of MYC in BC tissues. (E) Correlation analysis of circRRM2 and MYC in BC tissues. (F-G) circRRM2 knockdown abolished the suppression of cell migration treated with miR-27b-3p/miR-31-5p inhibitor. BT-549 cells were transfected with miR-27b-3p (F) or miR-31-5p (G) inhibitor, and the scratch wound healing assay was performed to measure the ability of cell migration. The rescue assay was conducted by co-transfecting the circRRM2 plasmid. *P < 0.05, **P < 0.01, ***P < 0.001. Fig. S7. Prediction and prognostic value of target genes binding with both miR-27b-3p and miR-31-5p. Fig. S8. Prognostic value of target genes binding with both miR-27b-3p and miR-31-5p. *P < 0.05, **P < 0.01, ***P < 0.001. Fig. S9. The transwell assay in BC cell transfected with circRRM2 plasmid and IGF2BP1 siRNA. The transwell assay was performed to detect the rescue effect of overexpression of circRRM2 on IGF2BP1 knockdown in BT-549 (A) or MDA-MB-231 (B) cells. Fig. S10. Calibration plot of the nomogram to predict the probability of the OS in patients wi [file 12935_2023_2895_MOESM1_ESM.zip › 20230321-supplementary materials/20230104-supplementary materials/Supplementary figures/Figure S5.pdf]

**A**

MDA-MB-231

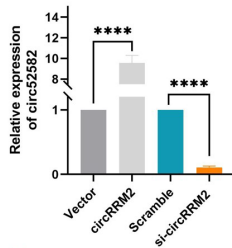**B**

BT-549

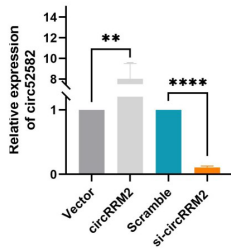**C**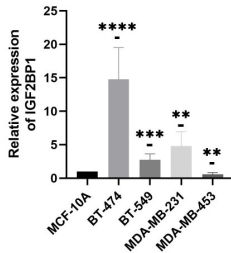**D**

Relative expression of MYC

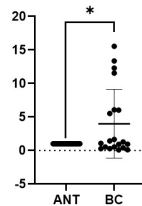**E**

Relative expression of MYC

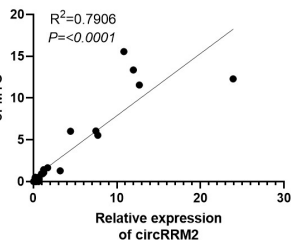**F**

BT-549

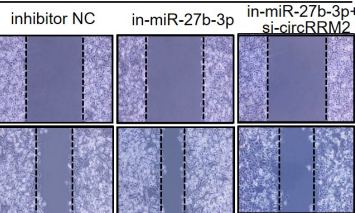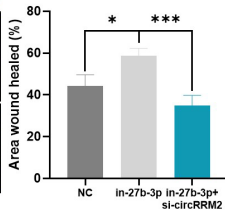**G**

BT-549

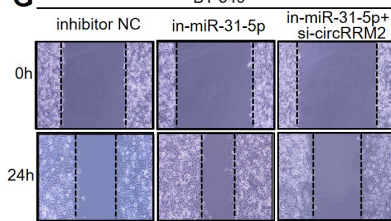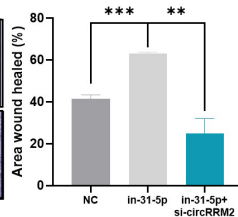

Supplement: Supplementary file 1 — Additional file 1: Fig. S1. Analysis of four candidate circRNAs and enrichment analysis of parent genes. Fig. S2. The mRNA levels of hsa_circ_0052582, hsa_circ_0058113, hsa_circ_0058148, and hsa_circ_0060551 in patients with BC from the GEO dataset (GSE111504). Fig. S3. The mRNA levels of hsa_circ_0052582, hsa_circ_0058113, hsa_circ_0058148, and hsa_circ_0060551 in patients with BC at different T stages from the GEO dataset (GSE111504). Fig. S4. The mRNA levels of hsa_circ_0052582, hsa_circ_0058113, hsa_circ_0058148, and hsa_circ_0060551 in patients with BC at different N stages from the GEO dataset (GSE111504). Fig. S5. CircRNA-miRNA-mRNA ceRNA network. Fig. S6. Expression level of circRRM2/IGF2BP1/MYC in BC and the migration phenotype of miR-27b-3p/miR-31-5p inhibitor in BC cells. Overexpression (A) or knockout (B) efficiency of circRRM2 in BT-549 and MDA-MB-231 was verified by RT qPCR. (C) The levels of IGF2BP1 in BC cells. (D) Expression level of MYC in BC tissues. (E) Correlation analysis of circRRM2 and MYC in BC tissues. (F-G) circRRM2 knockdown abolished the suppression of cell migration treated with miR-27b-3p/miR-31-5p inhibitor. BT-549 cells were transfected with miR-27b-3p (F) or miR-31-5p (G) inhibitor, and the scratch wound healing assay was performed to measure the ability of cell migration. The rescue assay was conducted by co-transfecting the circRRM2 plasmid. *P < 0.05, **P < 0.01, ***P < 0.001. Fig. S7. Prediction and prognostic value of target genes binding with both miR-27b-3p and miR-31-5p. Fig. S8. Prognostic value of target genes binding with both miR-27b-3p and miR-31-5p. *P < 0.05, **P < 0.01, ***P < 0.001. Fig. S9. The transwell assay in BC cell transfected with circRRM2 plasmid and IGF2BP1 siRNA. The transwell assay was performed to detect the rescue effect of overexpression of circRRM2 on IGF2BP1 knockdown in BT-549 (A) or MDA-MB-231 (B) cells. Fig. S10. Calibration plot of the nomogram to predict the probability of the OS in patients wi [file 12935_2023_2895_MOESM1_ESM.zip › 20230321-supplementary materials/20230104-supplementary materials/Supplementary figures/Figure S6.pdf]

**A**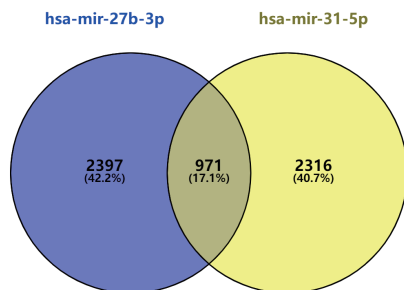**B**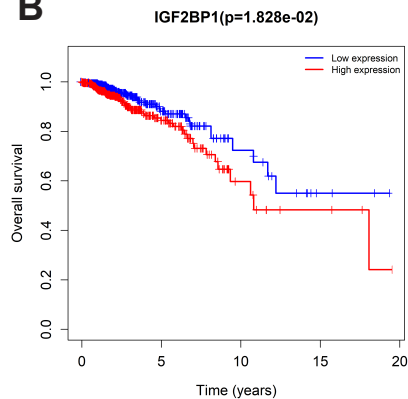**C**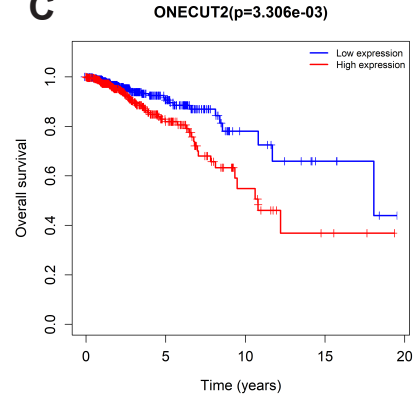**D**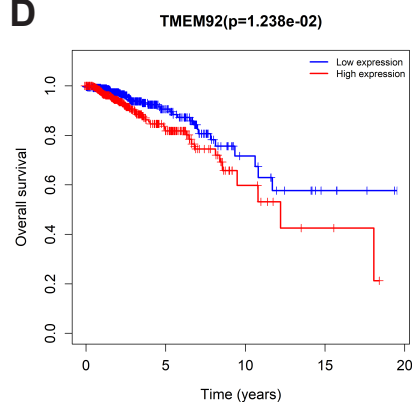**E**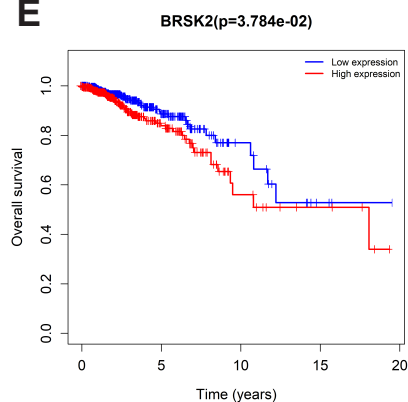**F**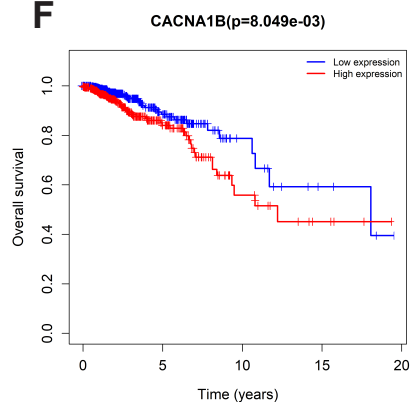**G**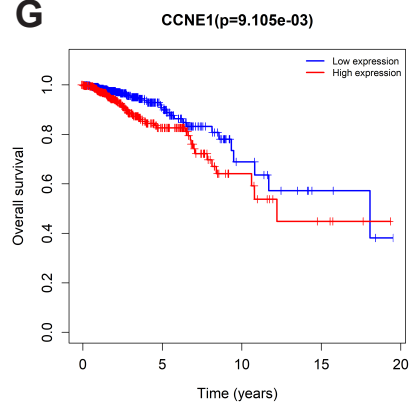**H**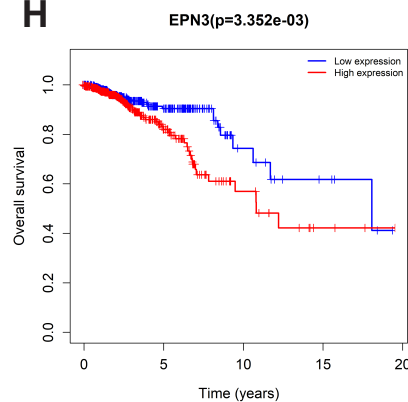**I**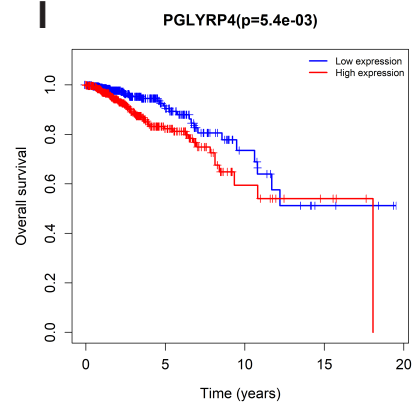

Supplement: Supplementary file 1 — Additional file 1: Fig. S1. Analysis of four candidate circRNAs and enrichment analysis of parent genes. Fig. S2. The mRNA levels of hsa_circ_0052582, hsa_circ_0058113, hsa_circ_0058148, and hsa_circ_0060551 in patients with BC from the GEO dataset (GSE111504). Fig. S3. The mRNA levels of hsa_circ_0052582, hsa_circ_0058113, hsa_circ_0058148, and hsa_circ_0060551 in patients with BC at different T stages from the GEO dataset (GSE111504). Fig. S4. The mRNA levels of hsa_circ_0052582, hsa_circ_0058113, hsa_circ_0058148, and hsa_circ_0060551 in patients with BC at different N stages from the GEO dataset (GSE111504). Fig. S5. CircRNA-miRNA-mRNA ceRNA network. Fig. S6. Expression level of circRRM2/IGF2BP1/MYC in BC and the migration phenotype of miR-27b-3p/miR-31-5p inhibitor in BC cells. Overexpression (A) or knockout (B) efficiency of circRRM2 in BT-549 and MDA-MB-231 was verified by RT qPCR. (C) The levels of IGF2BP1 in BC cells. (D) Expression level of MYC in BC tissues. (E) Correlation analysis of circRRM2 and MYC in BC tissues. (F-G) circRRM2 knockdown abolished the suppression of cell migration treated with miR-27b-3p/miR-31-5p inhibitor. BT-549 cells were transfected with miR-27b-3p (F) or miR-31-5p (G) inhibitor, and the scratch wound healing assay was performed to measure the ability of cell migration. The rescue assay was conducted by co-transfecting the circRRM2 plasmid. *P < 0.05, **P < 0.01, ***P < 0.001. Fig. S7. Prediction and prognostic value of target genes binding with both miR-27b-3p and miR-31-5p. Fig. S8. Prognostic value of target genes binding with both miR-27b-3p and miR-31-5p. *P < 0.05, **P < 0.01, ***P < 0.001. Fig. S9. The transwell assay in BC cell transfected with circRRM2 plasmid and IGF2BP1 siRNA. The transwell assay was performed to detect the rescue effect of overexpression of circRRM2 on IGF2BP1 knockdown in BT-549 (A) or MDA-MB-231 (B) cells. Fig. S10. Calibration plot of the nomogram to predict the probability of the OS in patients wi [file 12935_2023_2895_MOESM1_ESM.zip › 20230321-supplementary materials/20230104-supplementary materials/Supplementary figures/Figure S7.pdf]

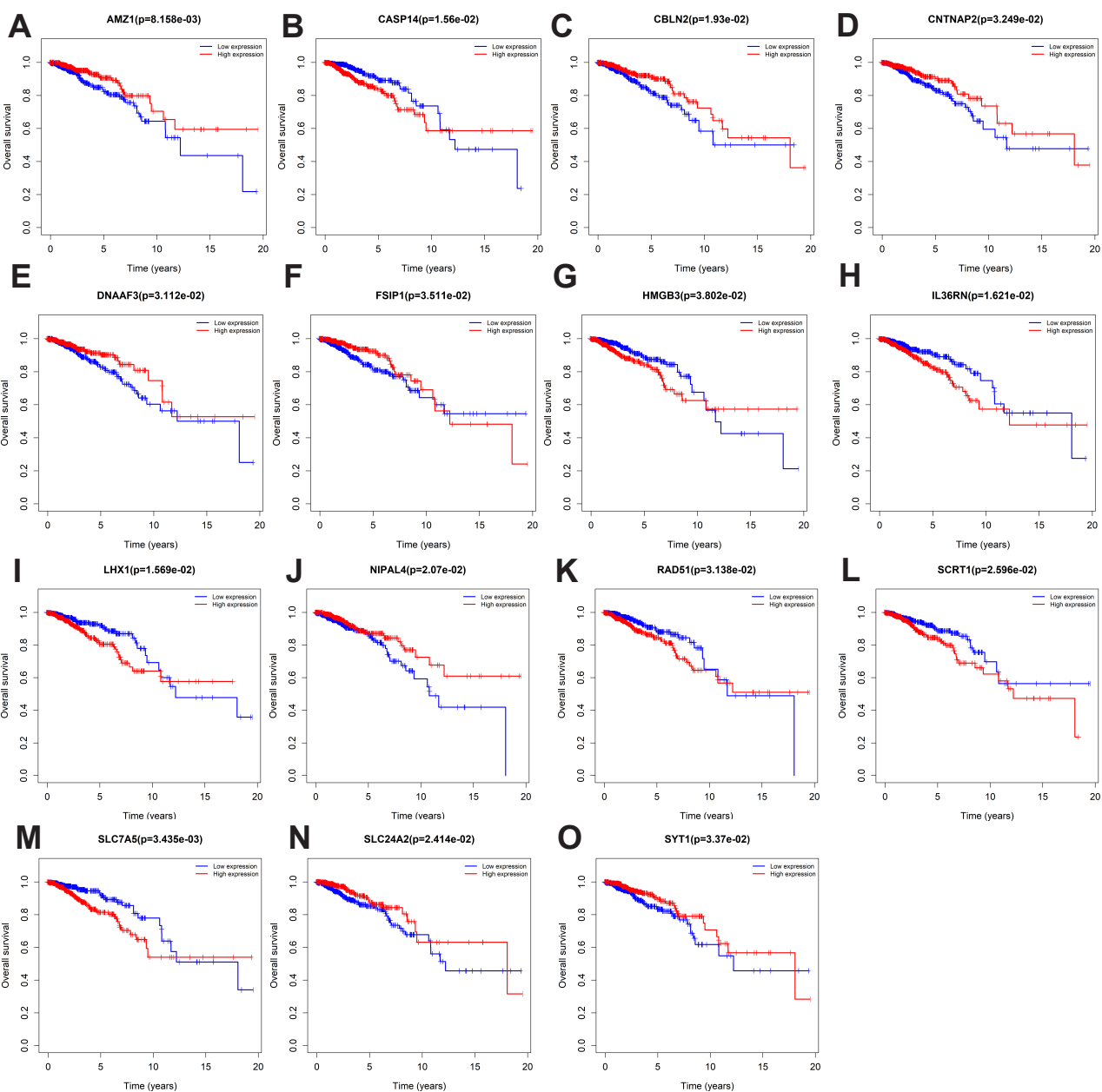

Supplement: Supplementary file 1 — Additional file 1: Fig. S1. Analysis of four candidate circRNAs and enrichment analysis of parent genes. Fig. S2. The mRNA levels of hsa_circ_0052582, hsa_circ_0058113, hsa_circ_0058148, and hsa_circ_0060551 in patients with BC from the GEO dataset (GSE111504). Fig. S3. The mRNA levels of hsa_circ_0052582, hsa_circ_0058113, hsa_circ_0058148, and hsa_circ_0060551 in patients with BC at different T stages from the GEO dataset (GSE111504). Fig. S4. The mRNA levels of hsa_circ_0052582, hsa_circ_0058113, hsa_circ_0058148, and hsa_circ_0060551 in patients with BC at different N stages from the GEO dataset (GSE111504). Fig. S5. CircRNA-miRNA-mRNA ceRNA network. Fig. S6. Expression level of circRRM2/IGF2BP1/MYC in BC and the migration phenotype of miR-27b-3p/miR-31-5p inhibitor in BC cells. Overexpression (A) or knockout (B) efficiency of circRRM2 in BT-549 and MDA-MB-231 was verified by RT qPCR. (C) The levels of IGF2BP1 in BC cells. (D) Expression level of MYC in BC tissues. (E) Correlation analysis of circRRM2 and MYC in BC tissues. (F-G) circRRM2 knockdown abolished the suppression of cell migration treated with miR-27b-3p/miR-31-5p inhibitor. BT-549 cells were transfected with miR-27b-3p (F) or miR-31-5p (G) inhibitor, and the scratch wound healing assay was performed to measure the ability of cell migration. The rescue assay was conducted by co-transfecting the circRRM2 plasmid. *P < 0.05, **P < 0.01, ***P < 0.001. Fig. S7. Prediction and prognostic value of target genes binding with both miR-27b-3p and miR-31-5p. Fig. S8. Prognostic value of target genes binding with both miR-27b-3p and miR-31-5p. *P < 0.05, **P < 0.01, ***P < 0.001. Fig. S9. The transwell assay in BC cell transfected with circRRM2 plasmid and IGF2BP1 siRNA. The transwell assay was performed to detect the rescue effect of overexpression of circRRM2 on IGF2BP1 knockdown in BT-549 (A) or MDA-MB-231 (B) cells. Fig. S10. Calibration plot of the nomogram to predict the probability of the OS in patients wi [file 12935_2023_2895_MOESM1_ESM.zip › 20230321-supplementary materials/20230104-supplementary materials/Supplementary figures/Figure S8.pdf]

**A**

BT-549

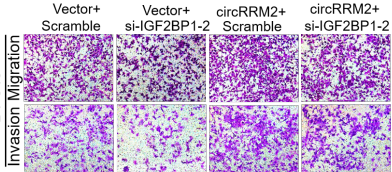

Relative cell numbers

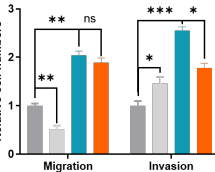**B**

MDA-MB-231

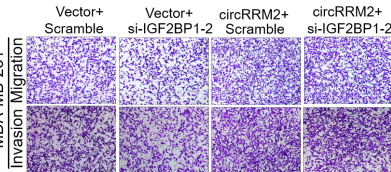

Relative cell numbers

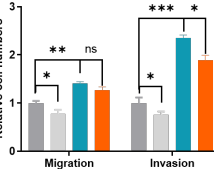

Supplement: Supplementary file 1 — Additional file 1: Fig. S1. Analysis of four candidate circRNAs and enrichment analysis of parent genes. Fig. S2. The mRNA levels of hsa_circ_0052582, hsa_circ_0058113, hsa_circ_0058148, and hsa_circ_0060551 in patients with BC from the GEO dataset (GSE111504). Fig. S3. The mRNA levels of hsa_circ_0052582, hsa_circ_0058113, hsa_circ_0058148, and hsa_circ_0060551 in patients with BC at different T stages from the GEO dataset (GSE111504). Fig. S4. The mRNA levels of hsa_circ_0052582, hsa_circ_0058113, hsa_circ_0058148, and hsa_circ_0060551 in patients with BC at different N stages from the GEO dataset (GSE111504). Fig. S5. CircRNA-miRNA-mRNA ceRNA network. Fig. S6. Expression level of circRRM2/IGF2BP1/MYC in BC and the migration phenotype of miR-27b-3p/miR-31-5p inhibitor in BC cells. Overexpression (A) or knockout (B) efficiency of circRRM2 in BT-549 and MDA-MB-231 was verified by RT qPCR. (C) The levels of IGF2BP1 in BC cells. (D) Expression level of MYC in BC tissues. (E) Correlation analysis of circRRM2 and MYC in BC tissues. (F-G) circRRM2 knockdown abolished the suppression of cell migration treated with miR-27b-3p/miR-31-5p inhibitor. BT-549 cells were transfected with miR-27b-3p (F) or miR-31-5p (G) inhibitor, and the scratch wound healing assay was performed to measure the ability of cell migration. The rescue assay was conducted by co-transfecting the circRRM2 plasmid. *P < 0.05, **P < 0.01, ***P < 0.001. Fig. S7. Prediction and prognostic value of target genes binding with both miR-27b-3p and miR-31-5p. Fig. S8. Prognostic value of target genes binding with both miR-27b-3p and miR-31-5p. *P < 0.05, **P < 0.01, ***P < 0.001. Fig. S9. The transwell assay in BC cell transfected with circRRM2 plasmid and IGF2BP1 siRNA. The transwell assay was performed to detect the rescue effect of overexpression of circRRM2 on IGF2BP1 knockdown in BT-549 (A) or MDA-MB-231 (B) cells. Fig. S10. Calibration plot of the nomogram to predict the probability of the OS in patients wi [file 12935_2023_2895_MOESM1_ESM.zip › 20230321-supplementary materials/20230104-supplementary materials/Supplementary figures/Figure S9.pdf]
